# Effectiveness and safety of selected traditional Chinese medicine injections in patients with combined diabetes mellitus and coronary heart disease: A systematic review and network meta-analysis of randomized clinical trials

**DOI:** 10.3389/fphar.2022.1060956

**Published:** 2023-01-09

**Authors:** Hailiang Shen, Ping Zhou, Luyao Shen, Chenhao Ju, Haixia Du, Xianguo Qu

**Affiliations:** ^1^ College of Basic Medical Science, Zhejiang Chinese Medical University, Hangzhou, China; ^2^ Department of Cardiology, Affiliated Hospital of Hangzhou Normal University, Hangzhou, China; ^3^ The Third School of Clinical Medicine, Henan University of Chinese Medicine, Zhengzhou, China; ^4^ Department of Acupuncture and Massage, Hangzhou Binjiang Hospital of Traditional Chinese Medicine, Hangzhou, China; ^5^ College of Life Science, Zhejiang Chinese Medical University, Hangzhou, China; ^6^ Affiliated Hangzhou First People’s Hospital, Zhejiang University School of Medicine, Hangzhou, China

**Keywords:** traditional Chinese medicine injection, diabetes mellitus, coronary heart disease, network meta-analysis, systematic review

## Abstract

**Background:** In view of the high morbidity and mortality of Diabetes mellitus—Coronary heart disease (DM-CHD) in diabetics, the combination therapy of traditional Chinese medicine injections (TCMIs) and conventional therapy (CT) is receiving extensive attention. Therefore, the effectiveness and security of conventional therapy with traditional Chinese medicine injections in the therapy of diabetes mellitus—coronary heart disease were compared by systematical review and network meta-analysis.

**Methods:** According to the preset inclusion criteria and exclusion criteria, we searched seven electronic literature databases from their inception to JAN 5,2022, to obtain the relevant RCT literature on the therapy of diabetes mellitus—coronary heart disease with traditional Chinese medicine injections. Two researchers independently reviewed the papers, two other researchers worked in extracting data and quality assessment of the included literature. The primary outcomes were total effective rate. The secondary outcomes included electrocardiogram (EGG)effective rate, the effective rate of angina pectoris, fasting blood glucose (FBG), 2-h postprandial blood glucose (PBG), hemoglobinA1c (HbA1c), total cholesterol (TC) and triglycerides (TG), high-density lipoprotein (HDL), low-density lipoprotein (LDL), frequency of angina pectoris, and duration of angina pectoris. We adopted stata16.0 software for the systematic review and network meta-analysis.

**Results:** A total of 53 trials involved 4,619 patients and one of the following 16 traditional Chinese medicine injections: Danhong, Danshen, Gualoupi, Gegen, Chuanxiongqin, Danshenchuanxiongqin, Shenmai, Shenqi, Xixin, Xuesaitong, Shuxuetong, Guanxinning, Kudiezi, Ciwujia, Xingding, Shuxuening. The meta-analysis revealed that Chuanxiongqin injection was superior to all other therapies in improving the total effective rate, [vs. conventional therapy odds ratio (OR): 14.52, 95% confidence interval (CI): 4.13–51.02], vs. Xuesaitong injection (odds ratio: 7.61, confidence interval: 1.25–46.40), and vs. Danshenchuanxiongqin injection (odds ratio: 3.98, confidence interval: 1.03–15.28)]. Xixin injection + conventional therapy was superior to conventional therapy only for electrocardiogram effective rate (odds ratio: 5.44, confidence interval: 1.55–19.18). Shenmai injection + conventional therapy was superior to conventional therapy in effective rate of angina (odds ratio: 11.05, confidence interval: 2.76–44.28). There was not different significantly in the comparisons of frequency of angina pectoris and duration of angina pectoris, we considered that this may be due to the lack of sufficient data. As most of the included RCTs did not monitor Adverse Events, the safety of those traditional Chinese medicine injections remains to be further explored.

**Conclusion:** Basing on our study, traditional Chinese medicine injections combined with conventional therapy takes important role in the treatment of diabetes mellitus—coronary heart disease, and its curative effect is better than conventional therapy. Nevertheless, properly designed RCTs are required to validate our conclusions in the future.

**Systematic Review Registration**: [https://inplasy.com/inplasy-2021-12-0125/], identifier [INPLASY2021120125].

## Introduction

Diabetes mellitus (DM) is a disease characterized by hyperglycemia caused by metabolic disorders. It is one of the most important non-communicable diseases threatening human health at present (Petersmann et al., 2019). According to the latest data from the International Diabetes Federation (IDF), there were 537 million diabetic patients worldwide in 2021. It is estimated that the number of people with diabetes will increase by 46%, reaching 780 million by 2045 (Saeedi et al., 2019). The latest epidemiology survey of diabetes in China shows that the prevalence of diabetes in people aged 18 and over is 12.4%, so it is estimated that about 150 million people with diabetes are diabetics (Wang et al., 2021a). Coronary atherosclerotic heart disease (CHD) is one of the common complications of DM, which is a kind of heart disease caused by coronary atherosclerotic plaque leading to vascular stenosis or even obstruction, myocardial ischemia, hypoxia and necrosis (Viigimaa et al., 2020). Data show that diabetic patients are 4 times more likely to suffer from cardiovascular diseases than non-diabetic patients (Lodha et al., 2018). In addition, compared with non-diabetic patients, diabetes mellitus coronary heart disease (DM-CHD) has early onset, complex condition, difficult treatment and poor prognosis (Yu and Shao, 2021).

At present, the conventional treatments of DM-CHD include angiotensin-converting enzyme inhibitors (ACEI), angiotensin receptor blockers (ARB), statins to improve blood supply, blood glucose control and reduce the risk of complications (Yang et al., 2019; Wang et al., 2021b). However, these strategies require long-term treatment and have many adverse reactions and side effects, such as hyperkalemia, arrhythmia, deterioration of renal function, *etc.* (Saedder et al., 2014; Yang et al., 2017). Therefore, it is urgent to explore other potential and effective interventions for the treatment of DM-CHD.

In the theory of traditional Chinese medicine (TCM), diabetes and coronary heart disease belong to the category of “chest pain” and “diabetes” (Wang et al., 2019). With thousands of years of history and practical experience, TCM has accumulated rich experience in the cognition and treatment of DM-CHD, and has created many effective therapeutic methods, and TCM has been widely used as complementary and alternative approach to the treatment and prevention of cardiovascular diseases (Hao et al., 2017). Traditional Chinese medicine injections (TCMIs), as one of the methods of TCM for the treatment of DM-CHD, has been widely used in clinical practice (Zhang et al., 2008; Wang et al., 2017; Zhou, 2017). The main TCMIs include Danhong injection, Danshen injection, Gegen injection, Shenmai injection, Gualoupi injection, Chuanxiongqin injection. This study systematically evaluated the above TCMIs.

At present, there is little literature review and systematic review on TCMIs in the treatment of DM-CHD, and point to point RCTs comparison is also lacking. Through both direct and indirect comparison, the network meta-analysis can be compared and sorted to evaluate the safety and efficacy of different TCMIs in patients with DM-CHD, so as to select the best treatment (De Laat, 2017). Therefore, we conducted pairwise comparison and meta-analysis of related randomized controlled trials (RCTs) to compare the safety and efficacy of different TCMIs in patients with DM-CHD, so as to provide better help for clinical application.

## Methods

This study has been registered in the International Platform of Registered Systematic Review and Meta-Analysis Protocols (INPLASY), with the number INPLASY202120125. Our analysis was conducted based on the Preferred Reporting Item for Systematic and Meta-Analyses (PRISMA) guidelines for systematic review and meta-analysis (Hutton et al., 2015); see Supplementary material.

### Data sources and searches

We searched PubMed, Web of Science, Embase, China National Knowledge Infrastructure (CNKI) Database, Chinese Biological Medicine Literature Service System Database (CBM), China Science Journal Database, China Science and Technology Journal Database (VIP), and Wan-fang database (WF) to get relevant articles that we need. The included articles were published from the establishment of each database to JAN 5, 2022. The theme search used was the combination of medical subject headings terms and free text terms. Searched terms included (“Diabetes” or “Diabetes mellitus”) and (“Coronary Diseases” or “Coronary Heart Disease”) and (“Injection”) within the restriction limit of (“randomized controlled trial”). We also manually searched journals that may publish research related to our subject.

### Eligibility and exclusion criteria

The RCTs must meet the following requirements: 1) Participants: patients must meet the diagnostic criteria of coronary heart disease formulated by the Ministry of the health of the people’s Republic of China (Wang et al., 2018), and must meet the diagnostic criteria of diabetes published by WHO in 2019 (Kerner and Brückel, 2014). Ethnical, gender, age, and reasons for the disease are unlimited; 2) Interventions and comparisons: The control group was treated with traditional western medicine, including ACEIs or ARBs, antiplatelet drugs, hypoglycemic drugs, and statins. The treatment group was treated with one of the following 16 TCMIS based on routine western medicine treatment in the control group: Danhong injection, Danshen injection, Gualoupi injection, Gegen injection, Chuanxiongqin injection, Danshenchuanxiongqin injection, Shenmai injection, Shenqi injection, Xixin injection, Xuesaitong injection, Shuxuetong injection, Guanxinning injection, Kudiezi injection, Ciwujia injection, Xingding injection, Shuxuening injection (The details of TCMIs of all the included studies in the Supplementary Table S3); 3) Outcomes: the primary outcomes were total effective rate. The secondary outcomes included EGG effective rate, the effective rate of angina pectoris, fasting blood glucose (FBG), 2-h postprandial blood glucose (PBG), hemoglobinA1c (HbA1c), total cholesterol (TC), triglycerides (TG), high-density lipoprotein (HDL), low-density lipoprotein (LDL), frequency of angina pectoris, and duration of angina pectoris. Included trials needed to report at least one of these indicators; 4) Research design: randomized controlled trial.

Studies were excluded according to the following criteria: 1) Treatments in the control group contained other TCMIs, acupuncture, Chinese herbal medicine; 2) publications were duplicated; 3) control group was not set in the clinical study design; 4) the data was imperfect; 5) No relevant outcomes.

### Literature inclusion and data extraction

NoteExpress software was used for literature management, and the literature selection was independently completed by two researchers. Firstly, duplicate articles and systematic reviews of animal experiments were excluded. Articles were then preliminarily screened by reading the titles and abstracts according to the inclusion and exclusion criteria. The next step is to read the full text of the preliminarily screened articles and conduct further screening in strict accordance with the inclusion and exclusion criteria. If the results of the study are controversial at any stage, a third researcher would participate in the discussion. In addition, relevant data were extracted, including basic information (publication date, author’s name, title), detailed characteristics of included patients (sample size, gender, average age, course of treatment), intervention measures (drugs, dose), results (main results, secondary results) and RCT quality evaluation information. We also tried to contact the study authors by email, telephone or fax to obtain missing demographic information, such as the sample size, gender distribution, age, *etc.*

### Risk of bias assessment

We evaluated the quality of all the included RCT literature independently by two investigators according to the Cochrane risk of bias tool (Higgins et al., 2011), mainly based on the following items: method of random assignment, concealment of allocation scheme, blinding, completeness of outcome data, selective reporting of study results, and other sources of bias. Each item was classified as low, unclear, or high risk of bias. When any disagreement arose between the two investigators, it was resolved by a third investigator.

### Data synthesis and statistical analysis

Stata 16.0 software was used for meta-analysis of results statistics and data. For binary variables, results are shown as odds ratios (ORs) and corresponding 95% confidence intervals (95% CI). If the outcomes were continuous type variable, they would presented as mean differences (MDS) and 95% CI. In addition, if there are two or more studies for each outcome, a head-to-head pairwise meta-analysis was performed using a random-effects model. Using a frequency framework and random-effects model, different interventions were compared through network meta-analysis, and the results of the analysis were presented using the leaderboard. Surface plots under the cumulative ranking curve area (Sucra) were plotted to evaluate the ranking of each treatment measure in the systematic analysis according to the magnitude of the area under the cumulative ranking curve, which was equal to one when the treatment measure was best and equal to 0 when the treatment was worst.

In pairwise meta-analyses, clinical heterogeneity should be examined by calculating the I^2^statistic and taking the way of comparing data from potential effect modifiers. If I^2^was ≤50%, the heterogeneity was not obvious (Higgins et al., 2003). Inconsistency cannot be assessed because each network plot in our analysis was not circular. In the systematic analysis, we also assumed that the estimates of heterogeneity variance were consistent. As publication bias may occur due to the relatively large number of studies, we plotted funnel plots to determine whether there was publication bias. In addition, we also assessed the quality of included studies according to five aspects: study limitations, indirectness, inconsistency, imprecision, and publication bias.

## Results

### Literature selection

**FIGURE 1:**
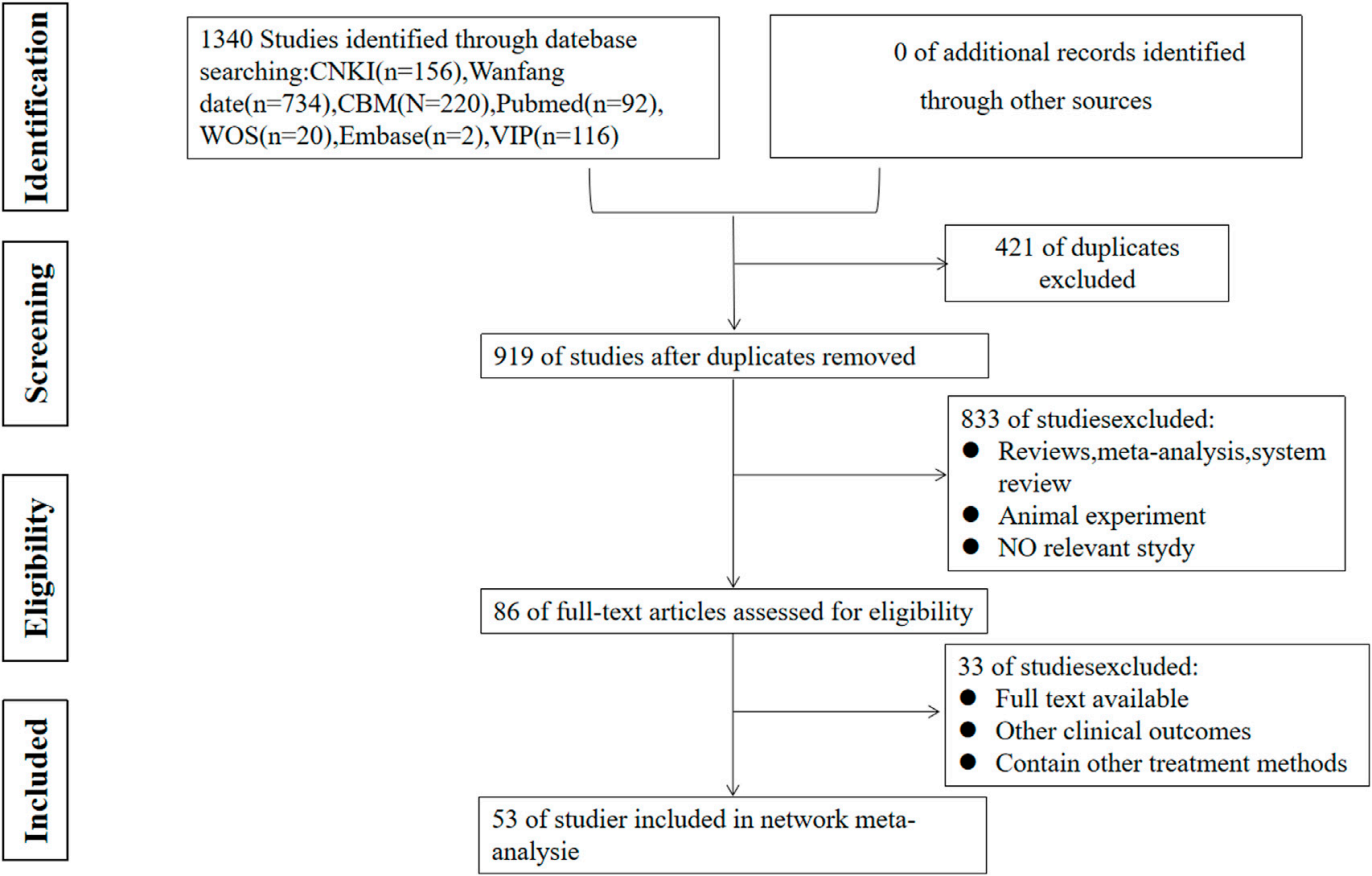
Flow chart of the study selection.

According to the established search strategy, we initially retrieved 1,340 articles, including 156 articles from CNKI, 734 articles from Wanfang, 220 articles from CBM, 116 articles from VIP, 92 articles from Pubmed, 20 articles from WOS, and two articles from Embase. A total of 919 articles were included after deleting duplicate articles. After carefully reading of the title and abstract of each artice, we excluded 783 artices that were not related to our study. In addition, we have carefully read the full-text of the remaining articles by Picos principles. Finally, we identified 53 RCTs, all of which have been published. In addition, the patient’s conditions and treatments must meet our pre-defined necessary conditions, and the outcomes in the literature must have at least one indicator as the primary outcome. The specific flow chart of the study is shown in Figure 1.

### Study characteristics

Among the 53 RCTs, a total of 4,619 patients were involved, and the patients participated in the trials were mostly middle-aged and elderly patients. All patients were treated with one of the 16 different TCMIs, as listed in the methods, in combination with conventional therapy. The main characteristics of all included studies are shown in Table 1.

**TABLE 1:** Characteristic of the articles included in the network meta-analysis.

Study ID	Sample size		Sex (M/F)		Age		Interventions		Course	Outcomes
	T	C	T	C	T	C	T	C	(days)	
Zhao and Han (2021)	50	50	25/25	23/27	62.45 ± 6.75	62.79 ± 7.02	Danhong injection 30 ml ivgtt qd + CT	CT	14	①③⑤⑥⑦
Lin and Hao (2020)	62	62	32/30	33/29	69.84 ± 5.58	69.95 ± 5.42	Guanxinning injection 20 ml ivgtt qd + CT	CT	14	①⑤⑦
Wang et al. (2021a)	50	50	25/25	23/27	62.45 ± 6.75	62.79 ± 7.02	Danhong injection 20 ml ivgtt qd + CT	CT	28	①③⑤⑥⑦
Sun et al., (2019)	40	40	18/22	20/20	45.23 ± 5.12	47.89 ± 6.89	Danhong injection 40 ml ivgtt qd + CT	CT	14	①②
Zhou (2017)	40	40	23/17	21/19	65.3 ± 6.7	67.1 ± 6.5	Gualoupi injection 8 ml ivgtt qd + CT	CT	28	⑤⑥⑦⑨⑩⑪
Fu (2017)	30	30	16/14	15/15	54.8	56.2	Danhong injection 40 ml ivgtt qd + CT	CT	14	④⑤⑥⑫⑬
Pei (2017)	31	31	17/14	16/15	57.6 ± 4.3	56.4 ± 3.7	Danshen injection 12 ml ivgtt qd + CT	CT	10	①⑫⑬
Chang (2017)	50	50	28/22	27/23	55.0 ± 3.8	55.0 ± 3.8	Chuanxiongqin injection 2 ml ivgtt qd + CT	CT	20	①⑤⑥⑦⑧⑨⑩⑪
Wang et al., 2017	48	48	NR	NR	NR	NR	Danhong injection 40 ml ivgtt qd + CT	CT	NR	②④
Jiao and Yang (2016)	40	20	28/12	13/7	67.50 ± 8.19	64.23 ± 9.82	Danhong injection 40 ml ivgtt qd + CT	CT	20	②
Guan (2015)	55	50	35/20	32/18	59 ± 4.2	58 ± 3.8	Danshenchuanxiongqin injection 10 ml ivgtt qd + CT	CT	14	①⑧⑨⑩⑪
Xia et al., (2015)	34	34	NR	NR	NR	NR	Danhong injection 40 ml ivgtt qd + CT	CT	14	①②③
Ji (2015)	45	45	32/13	34/11	46.3 ± 10.2	46.8 ± 10.6	Dengzhanxixin injection 40 ml ivgtt qd + CT	CT	28	①⑤⑥⑧⑨⑩⑪
Du and Li (2014)	30	28	NR	NR	NR	NR	Gegen injection 200 mg ivgtt qd + CT	CT	10	①②⑧⑨⑩⑪
Liu (2014)	60	60	NR	NR	NR	NR	Danhong injection 40 ml ivgtt qd + CT	CT	14	①⑧
Hu and Jia (2014)	38	38	20/18	21/17	54.72 ± 4.78	55.14 ± 4.63	Danshenchuanxiongqin injection10 ml ivgtt qd + CT	CT	14	①③⑧⑨⑩⑪⑫⑬
Fang and Wang (2014)	50	50	34/16	31/19	61.6 ± 4.7	60.4 ± 5.2	Danshenchuanxiongqin injection10 ml ivgtt qd + CT	CT	NR	①
Zhang et al. (2013)	50	50	27/23	36/14	55	54.5	Danhong injection 40 ml ivgtt qd + CT	CT	14	④⑤⑥⑫⑬
Tan and Li (2013)	40	40	NR	NR	NR	NR	Danhong injection 20 ml ivgtt qd + CT	CT	14	⑤⑥⑦⑧⑨⑩⑪
Lu (2012)	103	105	56/57	58/57	68.2 ± 4.2	69.1 ± 3.8	Guanxinning injection 30 ml ivgtt qd + CT	CT	14	①②
Yang (2012)	40	40	28/12	26/14	NR	NR	Danhong injection NR ivgtt qd + CT	CT	NR	①③⑧⑨⑩⑪
He (2012)	40	40	22/18	20/20	57.1 ± 7.9	52.1 ± 7.7	Danhong injection 40 ml ivgtt qd + CT	CT	14	①②
Gao (2011)	65	60	40/25	38/22	65.6 ± 6.8	64.3 ± 7.8	Danhong injection NR ivgtt qd + CT	CT	NR	④⑤⑥⑫⑬
Li and Jia (2011)	100	100	NR	NR	NR	NR	Guanxinning injection 30 ml ivgtt qd + CT	CT	14	②④
Fang (2011)	115	100	NR	NR	NR	NR	Shuxuetong injection 6 ml ivgtt qd + CT	CT	20	①⑧⑨⑩⑪
Dong (2009)	40	40	22/18	23/17	65	67.5	Shenqiong injection 100 ml ivgtt bid + CT	CT	14	②④
Wan (2009)	48	32	NR	NR	NR	NR	Danhong injection 20 ml ivgtt qd + CT	CT	14	①⑧⑨⑩⑪
Xing and Wang (2009)	65	60	40/25	38/22	65.6 ± 6.8	64.3 ± 7.8	Danhong injection 40 ml ivgtt qd + CT	CT	14	②③④⑤⑥⑫⑬
Xie et al., (2009)	30	30	18/12	19/11	67.36 ± 8.72	66.73 ± 7.89	Shenmaiinjection 30 ml ivgtt qd + CT	CT	14	①②⑤⑥
Wei and Zhou (2008)	49	48	28/21	27/21	61 ± 9.1	60 ± 8.9	Danshen injection 20 ml ivgtt qd + CT	CT	30	⑧⑨⑫
Wu et al., (2011)	57	57	NR	NR	NR	NR	Danshen injection 20 ml ivgtt qd + CT	CT	84	①⑤⑫⑬
Sun (2008)	32	32	19/13	18/14	62.90 ± 9.6	61.00 ± 10.5	Ciwujia injection 80 ml ivgtt qd + CT	CT	15	②④
Huang (2007)	32	32	20/12	19/13	59.6 ± 10.4	60.7 ± 11.3	Dengzhanxixin injection 20 ml ivgtt qd + CT	CT	14	①②⑧⑨⑩⑪
Wang and Zhang (2007)	30	30	16/14	18/12	62.15 ± 10.48	61.30 ± 9.89	Shuxuetong injection 6 ml ivgtt qd + CT	CT	14	③
Zeng et al., (2007)	28	28	17/11	15/13	57.7 ± 5.3	56.8 ± 7.3	Kudiezi injection 40 ml ivgtt qd + CT	CT	14	①②⑧⑨⑩⑪
Liao (2006)	50	50	32/18	34/16	65.3	64.8	Shenmaiinjection 40 ml ivgtt qd + CT	CT	15	①②
Du and Li (2006)	30	28	17/13	15/13	57.7 ± 5.3	56.8 ± 7.3	Gegen injection 400 mg ivgtt qd + CT	CT	14	①②⑧⑨⑩⑪
Zhang (2006)	20	20	10/10	12/8	63.5 ± 9.65	62.8 ± 11.5	Shuxuetong injection 6 ml ivgtt qd + CT	CT	14	①③⑤⑥⑦⑧⑨⑩⑪
Liu et al., (2005)	19	19	11/8	19/9	47	47.8	Shuxuening injection NR ivgtt qd + CT	CT	14	①③
Hou et al., (2003)	36	40	20/16	26/14	NR	NR	Ciwujia injection 40 ml ivgtt qd + CT	CT	14	①③
Li et al., (2003)	30	28	19/11	15/13	57.7 ± 5.3	56.8 ± 7.3	Xingding injection 20 ml ivgtt qd + CT	CT	14	①②③⑧⑨⑩⑪
Liu and Ma (2001)	32	20	18/14	12/8	NR	NR	Chuanxiongqin injection 0.1 g ivgtt qd + CT	CT	14	①③
Liu et al., (2001)	32	30	12/20	9/21	NR	NR	Shengmai injection 10 ml ivgtt qd + CT	CT	14	②③④⑤
Zhang (2005)	31	30	NR	NR	NR	NR	Dengzhanxixin injection 75 mg ivgtt qd + CT	CT	15	①③
Zhao et al., (2020)	36	36	21/15	22/14	53.2 ± 2.2	52.9 ± 2.1	Danhong injection 30 ml ivgtt qd + CT	CT	30	①
Zhang (2020)	52	52	30/22	28/24	51.6 ± 8.2	50.4 ± 9.5	Danshenchuanxiongqin injection 10 ml ivgtt qd + CT	CT	14	①⑧⑨⑩⑪
Fang and Li (2016)	46	45	29/17	31/14	53.81 ± 12.47	54.05 ± 12.86	Danshenchuanxiongqin injection10 ml ivgtt qd + CT	CT	14	①⑧⑨⑩⑪
Liu (2012)	36	36	13/23	15/21	47 ± 7.5	45 ± 8.5	Ciwujia injection 40 ml ivgtt qd + CT	CT	14	①
Deng and Gu (2020)	34	34	19/15	20/14	64.76 ± 5.15	66.27 ± 4.70	Danshenchuanxiongqin injectionNR ivgtt qd + CT	CT	NR	①⑧⑨⑩⑪
Wang and Kuang (2013)	20	20	11/9	10/10	60.2 ± 7.0	60.1 ± 6.8	Xuesaitong injection 400 mg ivgtt qd + CT	CT	15	①②③
Jia et al., (2016)	60	60	32/28	31/29	61.2 ± 3.12	60.4 ± 2.42	Danshen injection 200 mg ivgtt qd + CT	CT	10	②③④⑤
Wu et al., (2018)	48	42	30/18	NR	70.10 ± 5.3	67.70 ± 7.93	Shenmaiinjection 40 ml ivgtt qd + CT	CT	42	①⑧⑨⑩⑪⑫⑬
He et al., (2008)	30	20	17/13	12/8	NR	NR	Shuxuetong injection6ml ivgtt qd + CT	CT	14	②④⑤⑧⑨⑪⑫⑬

T, treatment group; C, control group; M, male; F, female; CT, conventional treatment; NR, not report; ivgtt, intravenous glucose tolerance test; qd, one time a day; ①Total effective rate; ②EGG, effective rate; ③adverse events; ④Effective rate of angina pectoris; ⑤fasting blood glucose; ⑥2-h postprandial blood glucose; ⑦hemoglobinA1c; ⑧total cholesterol; ⑨triglycerides; ⑩high-density lipoprotein; ⑪low-density lipoprotein; ⑫frequency of angina pectoris; ⑬duration of angina pectoris.

### Quality evaluation

**FIGURE 2:**
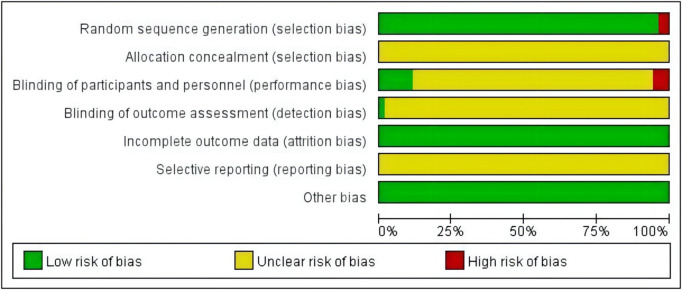
Summary of the risk of bias selection.

In terms of selective bias, the randomization of 11 articles was generated by a random number table, and therefore these articles were considered to be at low risk of bias. The two articles generated random sequences according to different clinical drugs, leading to higher risk of bias. The remaining RCTs used randomization only, without alternate the method of random assignment, and the risk of selection bias was considered unclear. In terms of performance bias, one study was double-blinded and five studies were all single-blinded, which were considered to be at low risk. However, three studies did not use blinding and were therefore rated as high risk. Other studies did not provide information on blinding, so performance bias was assessed as an unclear risk. Because there was incomplete information to assess the level of risk of detection bias, the risk levels were ambiguous. All articles are complete data and low risk. As the full trial protocol is not available, the risk of reporting bias was unclear. There were no other apparent biases in all studies and the risk was low. The summary of Risk of bias is shown in Figure 2, with green indicating low risk of bias, yellow indicating moderate risk of bias, and red indicating high risk of bias.

## Outcomes

### Total effective rate

**FIGURE 3:**
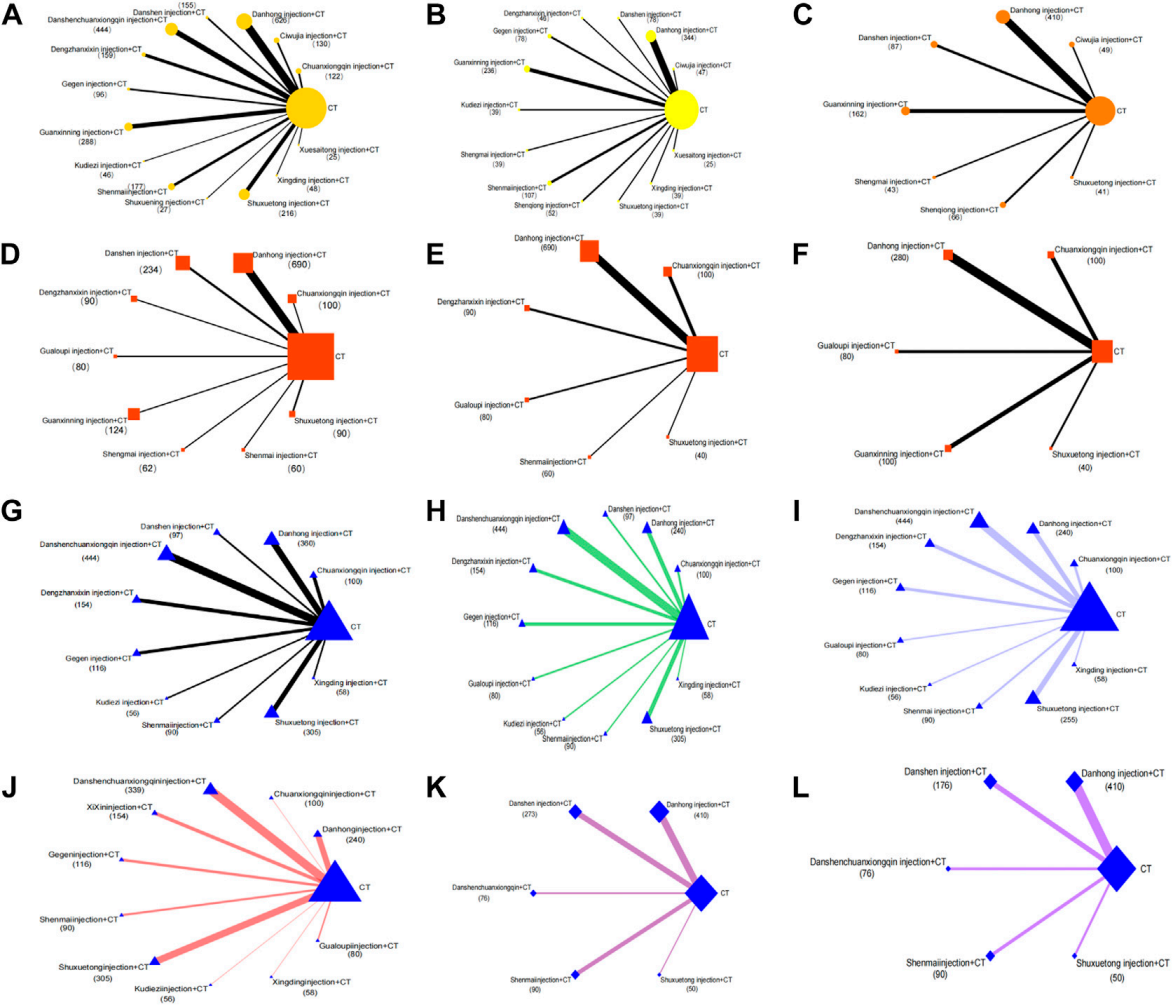
Network graphs of comparisons on different outcomes of treatments in different groups of patients with DM-CHD. **(A)** Total effective rate; **(B)** EGG effective rate; **(C)** Effective rate of angina pectoris; **(D)** FBG; **(E)** PBG; **(F)** HbA1c; **(G)** TC; **(H)** TG; **(I)** HDL; **(J)** LDL; **(K)** Frequency of angina pectoris; **(L)** Duration of angina pectoris.

**FIGURE 4:**
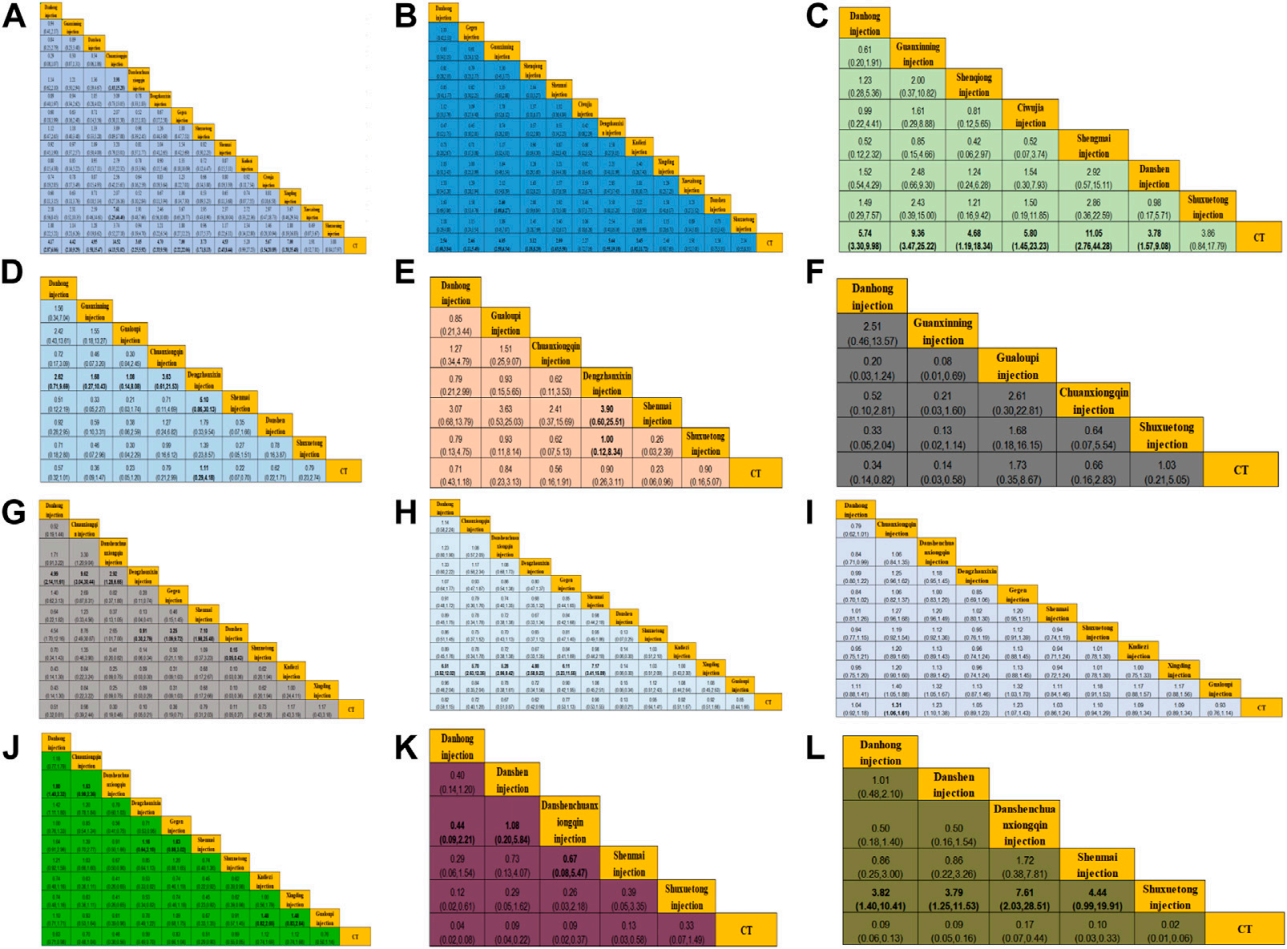
Final results of the systematic meta−analysis. **(A)** Total effective rate; **(B)** EGG effective rate; **(C)** Effective rate of angina pectoris; **(D)** FBG; **(E)** PBG; **(F)** HbA1c; **(G)** TC; **(H)** TG; **(I)** HDL; **(J)** LDL; **(K)** Frequency of angina pectoris; **(L)** Duration of angina pectoris.

In total, 37 RCTs were included involving studies of 14 treatments: DH + CT vs. CT (n = 9), DSCXQ + CT vs. CT (n = 6), XX + CT vs. CT (n = 3),SM + CT vs. CT (n = 3), GXN + CT vs. CT (n = 2), DS + CT vs. CT (n = 2), CXQ + CT vs. CT (n = 2), GG + CT vs. CT (n = 2), SXT + CT vs. CT (n = 2), CWJ + CT vs. CT (n = 2), KDZ + CT vs. CT (n = 1), XST + CT vs. CT (n = 1), SXN + CT vs. CT (n = 1), XD + CT vs. CT (n = 1). We plot a network plot of the results as shown in Figure 3A, with each row representing a head-to-head comparison type. The width of the line is proportional to the number of trials comparing the connected treatments. All types of TCMI + CT were superior to CT alone, and the difference was statistically significant in outcomes. The detailed comparative information is shown in Figure 4A: CT combined with the following TCMIs performed better than CT alone: Gegen injection (OR:7.00, CI: 2.22–22.06), Xingding injection (OR:7.00, CI: 1.38–35.48), Ciwujia injection (OR:5.67, CI: 1.54–20.89), Danshen injection (OR:4.95, CI: 1.58–15.47), Xixin injection (OR:4.70, CI: 2.33–9.50), Shenmai injection (OR:4.53,CI: 2.43–8.44), Guanxining injection (OR:4.42, CI: 2.10–9.29), Danhong injection (OR:4.17, CI: 2.87–6.06), Shuxuetong injection (OR:3.73, CI: 1.71–8.13), Danshenchuanxiongqin injection (OR:3.65, CI: 2.25–5.92). Among them, Chuanxiongqin injection was superior to all other treatment methods [vs. CT (OR: 14.52, CI: 4.13–51.02), vs. Xuesaitong injection (OR: 7.61, CI: 1.25–46.40), and vs. Danshenchuanxiongqin injection (OR: 3.98, CI: 1.03–15.28)].

### Electrocardiogram effective rate

For the EGG effective rate, there were a total of 21 RCTs involving 12 treatment regimens: DH + CT vs. CT (n = 6), SM + CT vs. CT (n = 3), GG + CT vs. CT (n = 2), GXN + CT vs. CT (n = 2), XX + CT vs. CT (n = 1), CWJ + CT vs. CT (n = 1), KDZ + CT vs. CT (n = 1), XD + CT vs. CT (n = 1), SXT + CT vs. CT (n = 1), XST + CT vs. CT (n = 1), DS + CT vs. CT (n = 1), SQ + CT vs. CT (n = 1). Its reticular diagram is shown in Figure 3B. In terms of ECG response rate, Xixin injection was superior to CT alone (OR:5.44, CI: 1.55–19.18), Danhong injection (OR:2.54, CI: 1.68–3.84), Gegen injection (OR:2.46, CI: 1.11–5.48), Guanxining injection (OR:4.05, CI: 2.58–6.34), Shenmai injection (OR:2.99, CI: 1.63–2.50), Kudieziinjection (OR:3.45, CI: 1.02–11.72).

### Effective rate of angina pectoris

There were a total of 11 RCTs on the effective rate of angina, involving seven treatment methods: DH + CT vs. CT (n = 5), GXN + CT vs. CT (n = 1), SQ + CT vs. CT (n = 1), CWJ + CT vs. CT (n = 1), SM + CT vs. CT (n = 1), DS + CT vs. CT (n = 1), SXT + CT vs. CT (n = 1). Its reticular diagram is shown in Figure 3C. Significant differences among the six treatments compared with CT alone are shown in Figure 4C: Shenmai injection + CTvs. CT (OR:11.05, CI: 2.76–44.28), Guanxining injection (OR: 9.36, CI: 3.47–25.22), Ciwujia injection (OR: 5.80, CI: 1.45–23.23), Danhong injection (OR:5.74, CI: 3.30–9.98), Shenqi injection (OR:4.68, CI:1.18–18.84), Danshen injection (OR:3.78, CI: 1.57–9.08).

## Fasting blood glucose

For the fasting blood glucose, its net diagram is shown in Figure 3D, involving a total of 17 RCTs and eight treatments, and all treatment comparisons compared were significant. Shenmai injection + CT had the best effect among all treatments, [vs. Danhong injection (MD: 2.62, CI: .71–9.96), vs. Guanxining injection (MD: 1.68, CI: .27–10.43), vs. Gualoupi injection (MD: 1.08, CI: .14–8.08), vs. Chuanxiongqin injection (MD: 3.63, CI: .61–21.53), and vs. XiXin injection (MD: 5.10, CI: .86–30.13)]. Compared with CT alone, the effect of Xixin injection was most significant.

## Postprandial blood glucose

There were 12 RCTs with six treatments, involving 2-h postprandial blood glucose, the reticular diagram of which is shown in Figure 3E. As shown in Figure 4E, all treatments were significant compared with CT alone. Xixin injection was superior to all other treatments, [vs. CT (MD:0.90, CI:0.26-3.31), vs. Danhong injection (MD:3.07, CI:0.68-13.79), vs. Gualoupi injection (MD: 3.63, CI: .53-25.03), vs. Chuanxiongqin injection (MD: 2.41, CI: .37-15.69), vs. Shenmai injection (MD: 3.90, CI:0.60–25.51) and vs. Shuxuetong injection (MD: 1.00, CI:0.12–8.34)].

## HemoglobinA1c

For glycated hemoglobin, a total of 7 RCTs were involved with five treatments, and their reticular diagram is shown in Figure 3F. CT combined with the following TCMIs: Danhong injection (MD:0.34, CI:0.14-.82), Guanxining injection (MD: .14, CI: .03-.58), Gualoupi injection (MD: 1.73, CI: .35–8.67), Chuanxiongqin injection (MD: .66, CI: .16–2.83), Shuxuetong injection (MD: 1.03, CI:0.21–5.05) were better than CT alone. Gualoupi injection had the best efficacy among all therapies.

## Total cholesterol

A total of 21 articles and 10 treatment measures involved total cholesterol, reticular diagram of which is shown in Figure 3G. All treatment comparisons were significantly different. Xixin injection, vs. Danhong injection (MD:4.99, CI:2.14-11.61), vs. Chuanxiongqin injection (MD: 9.62, CI: 3.04-30.44), and vs. Danshenchuanxiongqin injection (MD: 2.92, CI: 1.28-6.65); Danshen injection, vs.. Xixin injection (MD: .91, CI: .30-2.79), vs. Gegen injection (MD: 3.25, CI: 1.09–9.72), and vs. Shenmai injection (MD: 7.10, CI: 1.98–25.48). All 10 interventions were significantly better than CT alone, among which Kudiezi injection had the best effect compared with CT alone, and the detailed comparison information is presented in Figure 4G.

## Triglycerides

There were a total of 21 RCTs, of which 11 treatments involved triglycerides, and their reticular diagrams are shown in Figure 3H. All TCMIs were superior to CT alone. Among them, the best effect is Xinding Injection [vs. Danhong injection (MD: 6.51, CI: 3.52–12.02), vs. Chuanxiongqin injection (MD: 5.70, CI: 2.63–12.35), vs. Danshenchuanxiongqin injection (MD: 5.28, CI: 2.96–9.42), vs. Xixin injection (MD: 4.88, CI: 2.58–9.23), vs. Gegen injection (MD: 6.11, CI:3.23–11.55) and vs. Shenmai injection (MD: 7.17, CI: 3.41–15.09)].

## High-density lipoprotein

For HDL, a total of 19 RCTs and 10 treatments were involved, and their reticular diagrams are shown in Figure 3I. Chuanxiongqin injection was superior to CT alone (MD:1.31, CI: 1.06-1.61). Gualoupi injection was superior to other TCMIs, Gualoupi injection [vs. Chuanxiongqin injection (MD: 1.40, CI:1.05-1.88), vs. Danshenchuanxiongqin injection (MD: 1.32, CI:1.05-1.67), and vs. Gegen injection (MD: 1.32, CI:1.03–1.70)]. Additional detailed comparative information is presented in Figure 4I.

## Low-density lipoprotein

A total of 20 RCTs involving studies of 10 treatment methods, and the mesh diagram are shown in Figure 3J. There were significant differences among the treatments, Danshenchuanxiongqin vs. Danhong injection (MD: 1.80, CI: 1.40-2.32), vs. Chuanxiongqin injection (MD:1.53, CI: .99-2.36); Shenmai injection vs. Xixin injection (MD: 1.16, CI: .64-2.10), vs. Gegen injection (MD: 1.63, CI: .88-3.02). The comparison information is shown in Figure 4J.

### Frequency of angina pectoris

A total of 10 articles and five treatment measures related to the frequency of angina pectoris, and the network diagram is shown in Figure 3K. In the comparison of all therapies, Shenmai injection was significantly better than other therapies. Shenmai injection, vs. Danhong injection (MD: .44, CI: .09–2.21), vs. Danshen injection (MD: 1.08, CI: .20–5.84), vs. Danshenchuanxiongqin (MD: .67, CI:0.08–5.47). However, compared with CT alone, all TCMIs interventions had no obvious advantage in reducing the frequency of angina pectoris.

### Duration of angina pectoris

For the duration of angina pectoris, a total of 9 RCTs and five treatment methods were involved, and the network diagram is shown in Figure 3L. Among the five treatment methods, the most effective ones were Shuxuetong injection [vs. Danhong injection (MD: 3.82, CI: 1.40–10.41), vs. Danshen injection (MD: 3.79, CI: 1.25-11.53), vs. Danshenchuanxiongqin injection (MD: 7.61, CI: 2.03–28.51), and vs. Shenmai injection (MD: 4.44, CI: .99–19.91)]. However, compared with CT alone, all TCMIs were less effective in shortening the duration of angina pectoris than CT alone.

### Rank probabilities

**FIGURE 5:**
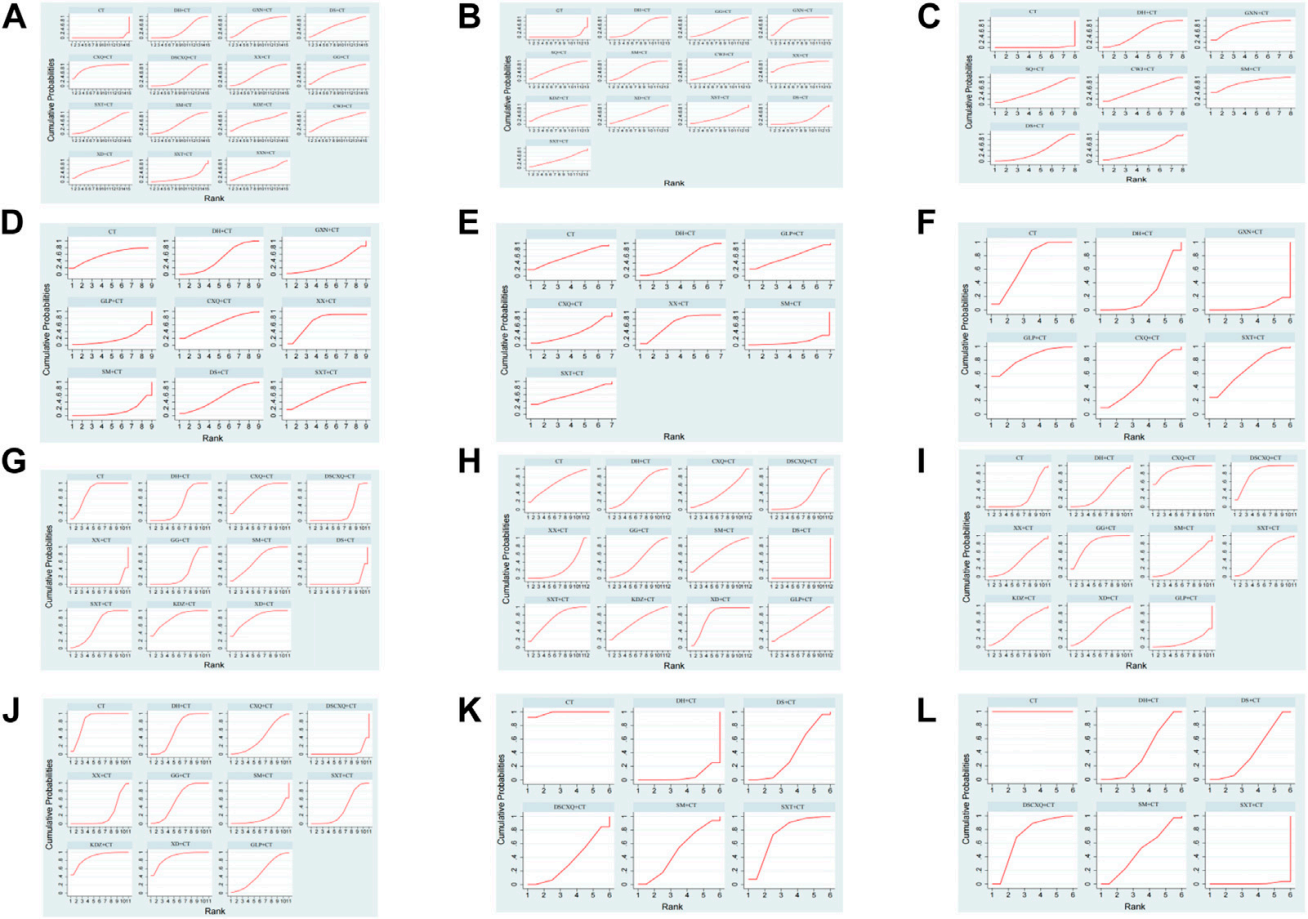
Ranking probabilities of comparable treatments. **(A)** Total effective rate; **(B)** EGG effective rate; **(C)** Effective rate of angina pectoris; **(D)** FBG; **(E)** PBG; **(F)** HbA1c; **(G)** TC; **(H)** TG; **(I)** HDL; **(J)** LDL; **(K)** Frequency of angina pectoris; **(L)** Duration of angina pectoris.

Figure 5 shows the overview of Bayesian ranking of the comparison treatments, with the vertical axis representing the cumulative probability and the horizontal axis representing the sequence of processing. The detailed sorting results are summarized in Table 2. Chuanxiongqin injection is most likely to rank first in overall effective rate (cumulative probability 34.6%), Xixin injection for ECG effective rate (44.0%), Shengmai injection for an effective rate of angina pectoris (44.3%), Xixin injection for FBG effective rate (38.6%), Shuxuetong injection for PBG effective rate (30.8%), Gualoupi injection for HbA1c effective rate (56.1%), Kudiezi injection for TC effective rate (33.0%), Xingding injection for TG effective rate (19.7%), Chuanxiongqin injection for HDL effective rate (54.1%), Kudiezi injection for LDL effective rate (45.0%). Detailed SUCRA value is shown in Table 2.

**TABLE 2:** SUCRA of different treatments for various outcomes.

TCMI	Total effective rate	EGG effective rate	Effective rate of angina pectoris	FBG	PBG	HbA1c	TC	TG	HDL	LDL	Frequency of angina pectoris	Duration of angina pectoris
CT	1.9	5.2	1.0	79.8	73.5	68.7	78.3	79.7	21.9	84.0	98.4	
DH	36.8	49.6	56.6	46.9	47.4	25.2	43.7	54.0	35.9	60.2	5.4	39.9
GXN	65.6	78.6	78.2	31.9	—	5.0	—	—	—	—	—	—
DS	58.3	24.2	37.5	52.8	—	—	6.0	—	—	—	38.5	40.1
CXQ	86.3	—	56.7	63.8	38.0	51.1	74.5	41.8	89.2	42.0	—	70.7
DSCXQ	37.5	—	—	—	—	—	23.9	29.9	82.3	4.7	34.8	—
XX	54.1	83.0	—	77.4	62.4	—	4.6	24.4	39.4	21.4	—	—
GG	64.4	48.8	—	—	—	—	31.2	46.7	81.0	59.5	—	—
SXT	44.7	43.0	40.7	64.3	60.8	66.8	60.7	71.2	52.2	37.4	73.8	0.9
SM	48.8	60.0	80.8	13.9	9.3	—	65.0	64.1	34.4	15.4	48.6	48.4
KDZ	59.2	65.1	—	—	—	—	81.1	64.9	50.8	88.1	—	—
CWJ	62.0	44.7	—	—	—	—	—	—	—	—	—	—
XD	61.9	48.9	—	—	—	—	81.0	66.2	50.3	88.1	—	—
XST	20.2	37.3	—	—	—	—	—	—	—	—	—	—
SXN	48.3	—	—	—	—	—	—	—	—	—	—	—
SQ	—	61.6	48.6	—	—	—	—	—	—	—	—	—
GLP	—	—	—	19.3	58.6	83.3	—	57.2	12.7	49.3	—	—

Grade, assessment.

We performed a GRADE assessment of the total effective rate using five downgrading factors: study limitations, inconsistency, indirectness, imprecision, and publication bias. The quality of evidence were graded as high, moderate,low, and very low, in which the rating of evidence quality for randomized controlled trials (RCTs) was preset as high, graded 1 as intermediate, 2 as low, and 3 as very low (Guyatt et al., 2011). Where XST + CT was downgraded by three for having a high risk of bias to be very low grade outside of this, the rest of the evidence grades were above low grade. Detailed GRADE grading is presented in Table 3.

**TABLE 3:** GRADE assessment for the total effective rate.

Comparison effect	Number of direct comparisons	Quality evaluation of evidence				Results summary			Evidence quality
		Limitation	Inconsistency	Indirect	Uncertainty	Publish bias	Treatment group	Control group	
DH + CT vs. CT	9	0	−1c	0	0	0	360/408	266/408	Moderate
GXN + CT vs. CT	2	0	0	0	−1e	−1e	157/167	131/167	Low
DS + CT vs. CT	2	0	0	0	−1e	−1e	84/88	71/88	Low
CXQ + CT vs. CT	2	0	0	0	−1e	−1e	77/80	58/80	Low
DSCXQ + CT vs. CT	6	−2a	0	0	0	0	248/275	196/275	Low
XX + CT vs. CT	3	−1b	−1d	0	0	0	94/107	65/107	Low
GG + CT vs. CT	2	0	0	0	−1e	−1e	56/60	40/60	Low
SXT + CT vs. CT	2	-	0	0	−1e	−1e	125/135	91/120	Low
SM + CT vs. CT	3	0	−1d	0	0	0	108/128	69/128	Moderate
KDZ + CT vs. CT	1	-	0	0	−1e	−1e	26/28	20/28	Low
CWJ + CT vs. CT	2	0	0	0	−1e	−1e	73/76	61/76	Low
XD + CT vs. CT	1	-	0	0	−1e	−1e	28/30	20/30	Low
XST + CT vs. CT	1	−1b	0	0	−1e	−1e	14/20	11/20	Very Low
SXN + CT vs. CT	1	0	0	0	−1e	−1e	16/19	11/19	Low

- indicates no evaluation; 0 indicates no degradation; - one indicates one level reduction; - two indicates two levels reduction.

a For inclusion in the study, there are two or more high risks of bias in terms of randomization, blinding, allocation concealment, integrity of outcome data or selective reporting.

b There is a high risk of bias in randomization, blinding, allocation concealment, integrity of outcome data or selective reporting for inclusion in the study.

c For inclusion in the study, 75% ≤ I2 ≤ 100%.

d For inclusion in the study, 50% ≤ I2 < 75%.

e The sample size of included studies is ≤100 or less than three included studies.

### Publication bias and sensitivity analysis

**FIGURE 6:**
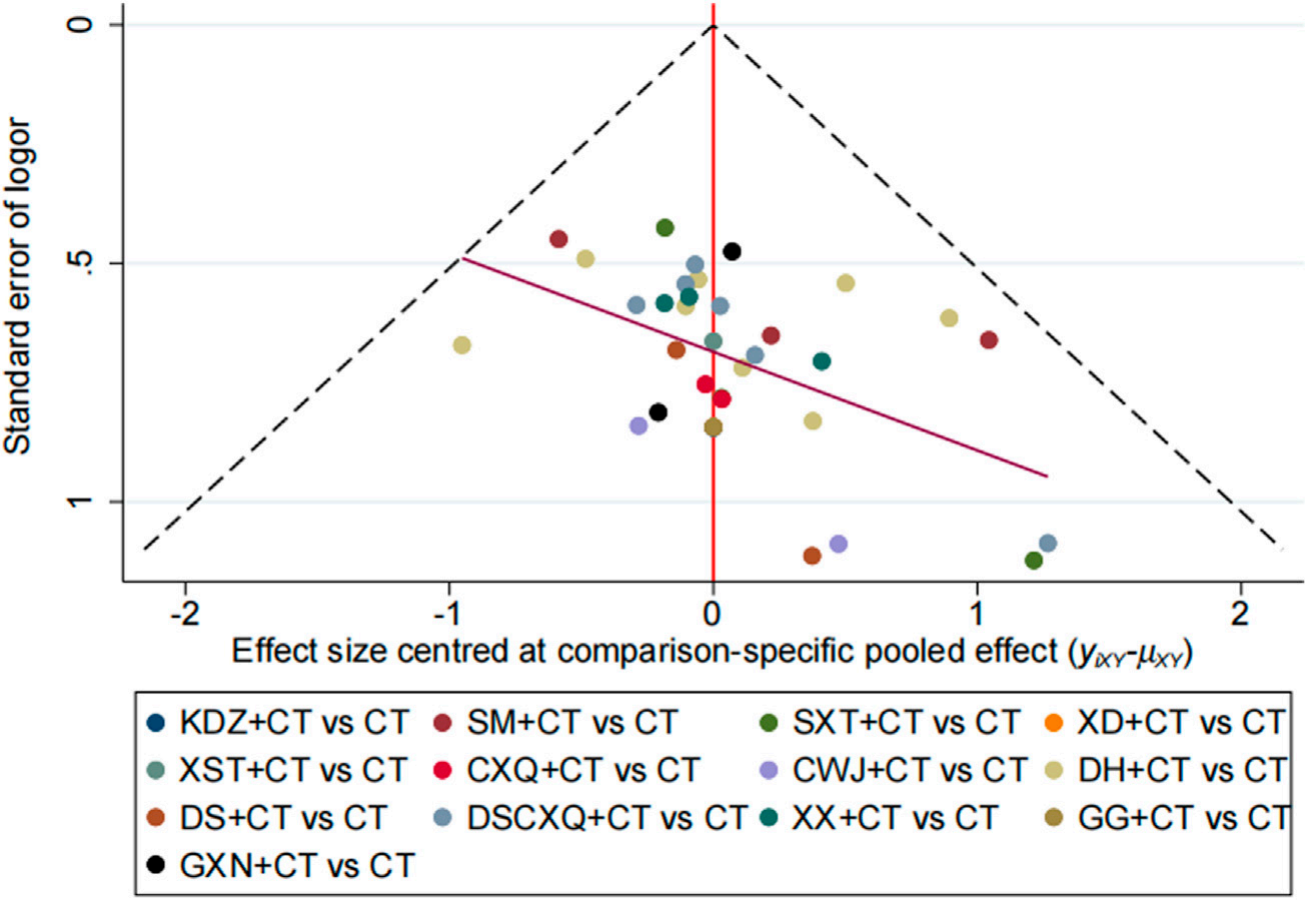
Funnel plot for total effective rate.

**FIGURE 7:**
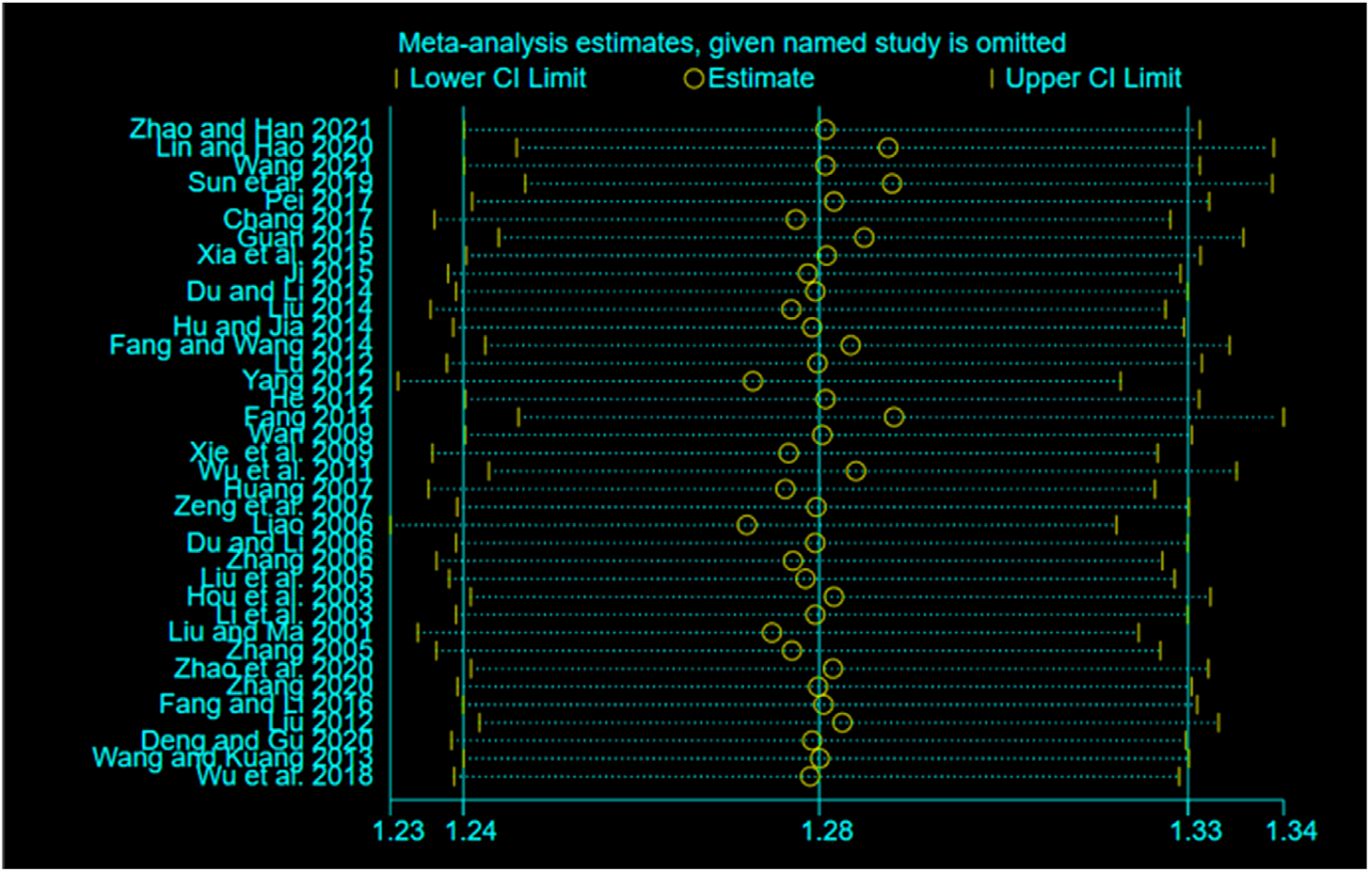
Sensitivity analysis results.

We plotted a funnel plot of the overall response rate to test for publication bias. As shown in Figure 6, we found that the various interventions were symmetrical along the centerline, and the angle between the centerline and the adjusted auxiliary lines was not large, suggesting less publication bias. The results of our concurrent sensitivity analysis are shown in Figure 7; while meeting the conditions of I^2^ < 50%, *p* > .1, and the point estimates of all outcomes are within 95% CIs of the pooled effect sizes. The results indicated that there were no findings to exclude, so we did not consider each study to have any bias in the results.

### Adverse events

14 records considered adverse reactions, 9 records did not clearly describe adverse reactions during medication, and 5 records recorded specific adverse reactions, such as headache, nausea, somnolence, and other outcomes as shown in Table 4. The incidences of adverse reactions were 7.47%, 7.89%, and 10.85% for Danhong injection, Danshenchuanxiong injection and CT alone, respectively.

**TABLE 4:** Adverse reactions of TCMIs.

No. of studies	Sample size	Treatments	Headache	Nausea	Drowsiness	Cardiopalmus	Hypoglycemia	Hyperglycemia
Zhao and Han 2021	50	Danhong	2	2	1		1	
Wang et al. (2021)	50	Danhong	2	2	1		1	
Xia et al., 2015	34	Danhong				1		
Hu and Jia 2014	38	Danshenchuanxiongqin		3				
Yang 2012	40	Danhong						
CT	212	CT	2	4	2	2	2	11

## Discussion

Abnormal glycolipid metabolism in diabetes can cause progressive damage to cardiomyocytes and activate cardiac fibroblasts, leading to myocardial interstitial fibrosis and progressive decline in cardiac function. Ang IV and its receptor AT_4_R overactivation in the renin-angiotensin system (RAAS) and cardiomyocyte autophagy abnormalities may be important mechanisms by which diabetes leads to myocardial injury and ventricular remodeling (Zhang et al., 2021). Meanwhile diabetes can cause pathological cardiac remodeling and heart failure by elevating the protein expression and activity of ADAM17, affecting ADRA1A regulation and AMPK signaling, and increasing myocardial fibrosis and cardiomyocyte apoptosis (Xue et al., 2022). Diabetes can aggravate coronary atherosclerosis through abnormal glycolipid metabolism, cardiomyocyte death, oxidative stress, and other pathways (Ritchie and Abel 2020), triggering coronary heart disease, which has become one of the important complications of diabetes. DM-CHD often leads to polyvascular, calcified, diffuse lesions in patients (Naito and Miyauchi, 2017), aggravates mortality of patients, and greatly affects patients’ mental health and quality of life. Despite significant progress in basic research, drugs, and interventions, the level of clinical efficacy and prognosis of DM-CHD remain unsatisfactory (Hinnen and Kruger, 2019). TCM has a long history in the treatment of the DM-CHD. In recent decades, many TCMIs with remarkable efficacy have been discovered in clinical practice, and TCMIs are playing an increasingly important role in the treatment of DM-CHD.

Danshen injection is extracted from Danshen (Salvia miltiorrhiza Bunge, Lamiaceae, Salviae miltiorrhizae radix et rhizoma), Danhong injection is extracted from Danshen and Honghua. And the main active components are danshensu, protocatechuic aldehyde, safflflower yellow A, and salvianolic acid (Jiang et al., 2015; Liu et al., 2019). Danshen injection and Danhong injection could activate blood circulation and remove stasis, widely used in the treatment of DM-CHD with blood stasis syndrome. Chuanxiongqin, Danshenchuanxiongqin, Guanxinning, Shuxuetong and Xixin injection have the same efficacy and are also used for treatment DM-CHD with the syndrome of chest pain and stuffiness, palpitations, dark purple tongue, etc (Li et al., 2022). Gegen, Shuxuening and Xingding injection play an important role in dilating coronary artery and improving myocardial ischemia (Fan et al., 2022). And there are used for treatment ischemic coronary heart disease and angina pectoris. Gualoupi and Kudiezi injection have the effect of Regulating Qi to dissipate blood stasis and phlegm, widely used in the treatment of DM-CHD with blood stasis and sputum dampness syndrome. Shenmai, Shenqi and Ciwujia injection are used for treatment DM-CHD with deficiency of vital qi.

The systematical analysis approach was taken to compare the efficacy and safety of different TCMI treatments in patients with DM-CHD. A total of 53 RCTs and 4,619 patients were included in this study, and we evaluated 16 treatment methods from 12 indicators involving total effective rate, EGG effective rate, the effective rate of angina pectoris, FBG, PBG, HbA1c, TC, TG, HDL, LDL, frequency of angina pectoris, duration of angina pectoris. The results indicated that all kinds of TCMIs + CT were more effective in the treatment of DM-CHD than CT alone.

In terms of primary outcomes included the total effective rate, EGG effective rate and effective rate of angina pectoris. The results suggested that all types of TCMIs + CT were superior to CT alone, and results were significantly different. In terms of total effective rate, Chuanxiongqin injection SUCRA had the largest area and ranked first, indicating Chuanxiongqin injection had the best effect among all therapies and was the best treatment for DM-CHD. However, Xixin injection and Shenmai injection play important roles in improving ECG and alleviating angina pectoris in DM-CHD patients respectively. Studies have found (Wang et al., 2020; Ye et al., 2021) that Xixin injection can significantly improve cardiac function by expanding coronary artery, increasing coronary flow, reducing cardiac load and myocardial gas consumption, improving ST segment and changing T wave from low to upright in patients’ ECG. Qi et al. (Qi et al., 2015) suggested that Shenmai injection can reduce the activities of serum creatine kinase (CK) and lactate dehydrogenase (LDH), resist ischemia-reperfusion injury, and protect myocardium from myocardial injury, and alleviate the symptoms of angina pectoris, which was also confirmed in our study.

The secondary outcomes included FBG, PBG, HbA1c, TC, TG, HDL, LDL, frequency of angina pectoris, and duration of angina pectoris. TCMIs were significantly better than CT alone in FBG, PBG, HbA1c, TC, TG, HDL and LDL, and SCURA ranking was higher than CT. Among them, Xixin injection ranked first in FBG and PBG, indicating that its curative effect in improving FBG and PBG indexes was significantly higher than that of other therapies. Kudiezi injection ranked first in TC and LDL as the best choice. Gualoupi injection, Xingding injection, and Chuanxiongqin injection play important roles in HbA1c, TG, and LDL, respectively, and had significant advantages compared with other therapies. Experiments showed that Danhong injection can reduce the blood lipid level, inhibit the expression of MMP9, prevent poor myocardial remodeling, reduce myocardial fibrosis and improve myocardial function (Chen et al., 2016). Danshen injection can improve the normalization of hemodynamic parameters, ventricular mass and cardiac functions (Zhang et al., 2013). Zhang (Zhang, 2016) believed that Shuxuetong injection can reduce erythrocyte aggregation, improve platelet activation function and alleviate myocardial injury. By reducing myocardial injury and protecting cardiac function, it can slow down the occurrence of angina pectoris. However, in our study, Danhong injection, Danshen injection and Shuxuetong injection had no significant advantages compared with CT alone in reducing the frequency and duration of angina pectoris, we speculated that this was caused by small sample size at that time.

The safety is crucial to TCMIs, and we also considered the safety in the test. A total 14 RCT in our study considered safety issues, 9 articles did not clearly describe adverse reactions during medication, 5 records described specific adverse reactions, and most did not report any ADRs. The specific adverse reactions were nausea, cardiopalmus, and drowsiness. Aizziness and hypoglycemia were reported occasionally. Although these ADRs could be effectively relieved by symptomatic treatments, clinicians should keep this in mind when prescribing TCMI treatments. In our comparisons, Danshenchuanxiongqin injection had the worst safety profile.

In conclusion, the efficacy of TCMIs + CT was better than that of CT alone in the treatment of DM-CHD. In combination therapy, Chuanxiongqin injection is the first choice to improve the effective rate, XiXin injection had the best curative effect when measuring by FBG and PBG, and Kudiezi injection can improve TC and LDL.

## Limitations

Our study had several limitations. First, no direct comparison between TCMIs were found between most treatments, and the most direct evidence was derived from one trial in the present network. Second, despite our best efforts, the quality of the included RCTs was low. Although the patients in the trials were randomized, only some of the 53 RCTs described specific randomization methods, such as random number table. And configuration concealment of some articles was unknown. Third, some trials were low sample size tests with positive findings and are particularly prone to various biases.

Due to the unsatisfactory quality of the included studies, based on our findings, we put forward the following two suggestions for further studies of TCMI-CT in the treatment of DM-CHD: 1) Prospective registration of large clinical trials in recognized clinical trial registration platform; 2) The trial protocol should be rigorously designed in terms of randomization, allocation, concealment, and blinding; 3) Design high-quality, large sample randomized controlled trial.

## Conclusion

In our study, we revealed that TCMIs had positive effects on patients with DM-CHD, but there was no significant difference in the frequency of angina pectoris, duration of angina pectoris. In addition, XiXin injection had the best curative effect when measured by FBG and PBG, and Kudiezi injection can improve TC and LDL. At the same time, we also discovered that Danshenchuanxiongqin injection has side effects, which should be paid more attention in clinical applications.

Nevertheless, on account of some limitations, more clinical studies with well-designed, reasonable samples and good method quality are needed to test and verify our results in the future.

## Data Availability

The original contributions presented in the study are included in the article/Supplementary Materials, further inquiries can be directed to the corresponding authors.
