# Supplementary material for: Effectiveness and safety of selected traditional Chinese medicine injections in patients with combined diabetes mellitus and coronary heart disease: A systematic review and network meta-analysis of randomized clinical trials
Source: Front Pharmacol. 2023 Jan 9;13:1060956. doi: 10.3389/fphar.2022.1060956 (PMC9868408; doi:10.3389/fphar.2022.1060956)
Supplement: Supplementary file 1 [file DataSheet1.doc]

***Supplementary table S1*** The details of search terms and literature search strategy

Take searching PubMed as an example, the search terms and strategies are as follows:

| #1Coronary Diseases [MeSH Terms]  #2 Coronary Diseases [Title/Abstract]  #3 Coronary Heart Disease [MeSH Terms]  #4 Coronary Heart Disease [Title/Abstract]  #5 #1 OR #2 OR #3 OR #4    #6Diabetes[MeSH Terms]  #7Diabetes[Title/Abstract]  #8Diabetes mellitus[MeSH Terms]  #9Diabetes mellitus[Title/Abstract]  #10 #6 OR #7 OR #8 OR #9    #11Traditional Chinese medicine [MeSH Terms]  #12 Traditional Chinese medicine [Title/Abstract]  #13 Injection [MeSH Terms]  #14 Injection [Title/Abstract]  #15 Injectable [MeSH Terms]  #16 Injectable [Title/Abstract]  #17 #11 OR #12 OR #13 OR #14OR #15 OR #16    #18Randomized Controlled Trial [Publication Type]  #19Controlled Clinical Trial [Publication Type]  #20random* [All Fields]  #21 #18 OR #19 OR #20  #22 #5AND #10 AND #17 AND #21 |
| --- |

***Supplementary table S2*** The Jadad scores of all the included studies

| Study ID | randomization | allocation concealment | blinding method | Withdrawal | Total score |
| --- | --- | --- | --- | --- | --- |
| Zhao and Han 2021 | 2 | 1 | 1 | 1 | 5 |
| Lin and Hao 2020 | 1 | 1 | 0 | 1 | 3 |
| Wang 2021 | 2 | 1 | 0 | 1 | 4 |
| Sun et al. 2019 | 2 | 1 | 0 | 1 | 4 |
| Zhou 2017 | 1 | 1 | 2 | 1 | 5 |
| Fu 2017 | 1 | 1 | 1 | 1 | 4 |
| Pei 2017 | 1 | 1 | 1 | 1 | 4 |
| Chang 2017 | 1 | 1 | 1 | 1 | 4 |
| Wang et al. 2017 | 1 | 1 | 1 | 1 | 4 |
| Jiao and Yang 2016 | 1 | 1 | 1 | 1 | 4 |
| Guan 2015 | 0 | 1 | 1 | 1 | 3 |
| Xia et al. 2015 | 2 | 1 | 1 | 1 | 5 |
| Ji 2015 | 2 | 1 | 1 | 1 | 5 |
| Du and Li 2014 | 1 | 1 | 1 | 1 | 4 |
| Liu 2014 | 1 | 1 | 1 | 1 | 4 |
| Hu and Jia 2014 | 2 | 1 | 1 | 1 | 5 |
| Fang and Wang 2014 | 1 | 1 | 1 | 1 | 4 |
| Zhang 2013 | 1 | 1 | 1 | 1 | 4 |
| Tan and Li 2013 | 1 | 1 | 1 | 1 | 4 |
| Lu 2012 | 1 | 1 | 1 | 1 | 4 |
| Yang 2012 | 1 | 1 | 1 | 1 | 4 |
| He 2012 | 1 | 1 | 1 | 1 | 4 |
| Gao 2011 | 1 | 1 | 1 | 1 | 4 |
| Li and Jia 2011 | 1 | 1 | 1 | 1 | 4 |
| Fang 2011 | 1 | 1 | 1 | 1 | 4 |
| Dong 2009 | 1 | 1 | 1 | 1 | 4 |
| Wan 2009 | 1 | 1 | 1 | 1 | 4 |
| Xing and Wang 2009 | 1 | 1 | 1 | 1 | 4 |
| Xie et al. 2009 | 2 | 1 | 1 | 1 | 5 |
| Wei and Zhou 2008 | 1 | 1 | 1 | 1 | 4 |
| Wu et al. 2011 | 1 | 1 | 1 | 1 | 4 |
| Sun 2008 | 2 | 1 | 1 | 1 | 5 |
| Huang 2007 | 1 | 1 | 1 | 1 | 4 |
| Wang and Zhang 2007 | 1 | 1 | 1 | 1 | 4 |
| Zeng et al. 2007 | 1 | 1 | 1 | 1 | 4 |
| Liao 2006 | 1 | 1 | 1 | 1 | 4 |
| Du and Li 2006 | 2 | 1 | 1 | 1 | 5 |
| Zhang 2006 | 1 | 1 | 1 | 1 | 4 |
| Liu et al. 2005 | 1 | 1 | 1 | 1 | 4 |
| Hou et al. 2003 | 1 | 1 | 1 | 1 | 4 |
| Li et al. 2003 | 1 | 1 | 1 | 1 | 4 |
| Liu and Ma 2001 | 0 | 1 | 1 | 1 | 3 |
| Liu et al. 2001 | 1 | 1 | 1 | 1 | 4 |
| Zhang 2005 | 1 | 1 | 1 | 1 | 4 |
| Zhao et al. 2020 | 2 | 1 | 1 | 1 | 5 |
| Zhang 2020 | 1 | 1 | 1 | 1 | 4 |
| Fang and Li 2016 | 1 | 1 | 1 | 1 | 4 |
| Liu 2012 | 1 | 1 | 1 | 1 | 4 |
| Deng and Gu 2020 | 1 | 1 | 1 | 1 | 4 |
| Wang and Kuang 2013 | 1 | 1 | 1 | 1 | 4 |
| Jia et al. 2016 | 1 | 1 | 1 | 1 | 4 |
| Wu et al. 2018 | 2 | 1 | 1 | 1 | 5 |
| He et al. 2008 | 1 | 1 | 1 | 1 | 4 |

***Supplementary table S3*** Botanical drugs included and traditional effects of TCMIs

| TCMI | Source species | Main component | Properties | Merdians | Traditional efficacy |
| --- | --- | --- | --- | --- | --- |
| Danhong injection | Salvia miltiorrhiza | danshensu, protocatechuic aldehyde | Minor cold,Bitter | Liver,Heart | invigorating the blood circulatio |
|  | Carthamus tinctorius | safflflower yellow A, salvianolic acid | Warm,Pungent | Liver,Heart |  |
| Guanxinning injection | Salvia miltiorrhiza | danshensu, protocatechuic aldehyde | Minor cold,Bitter | Liver,Heart | invigorating the blood circulation |
|  | Conioselinum anthriscoides (H.Boissieu) Pimenov & Kljuykov | Tetramethylpyrazine | Warm, Pungent | Liver, guts |  |
| Gualoupi injection | Trichosanthes kirilowii Maxim | Trichosanthes kirilowii Peel Polysaccharide | Cold,Sweet,Slightly Bitter | Lung,Large Intestine,Stomach | promoting the circulation of qi |
| Danshen injection | Salvia miltiorrhiza | danshensu, protocatechuic aldehyde | Minor cold,Bitter | Liver,Heart | invigorating the blood circlation |
| Chuanxiongqin injection | Conioselinum anthriscoides (H.Boissieu) Pimenov & Kljuykov | tetramethylpyrazine | Warm, Pungent | Liver, guts | invigorating the blood circulation |
| Danshen Chuanxiongqin  injection | Salvia miltiorrhiza | danshensu, protocatechuic aldehyde | Minor cold,Bitter | Liver,Heart | invigorating the blood circulation |
|  | Conioselinum anthriscoides (H.Boissieu) Pimenov & Kljuykov | tetramethylpyrazine | Warm, Pungent | Liver, guts |  |
| Dengzhanxixin injection | Erigeron breviscapus (Vaniot) Hand.-Mazz. | Scutellarin, caffeoylquinic acid | Warm, Pungent, Slightly Bitter | Heart, Liver | invigorating the blood circulation |
| Gegen injection | Pueraria montana | puerarin | Sweet,Cold, Pungent | Lung, Stomach | nourishing yin |
| Shuxuetong injection | Pheretima | fibrinolytic protein, lumbrokinase | Cold,Salty | Bladder,Spleen,Liver | invigorating the blood circulation |
|  | Aulastomum gulo | Hirudin, | Cold,Salty | Bladder,Spleen,Liver |  |
| Shenqiong injection | Salvia miltiorrhiza | danshensu, protocatechuic aldehyde | Minor cold,Bitter | Liver,Heart | invigorating the blood circulation |
|  | Conioselinum anthriscoides (H.Boissieu) Pimenov & Kljuykov | tetramethylpyrazine | Warm, Pungent | Liver, guts |  |
| Shenmai injection | Panax ginseng | Ginsenosides, polysorbate | Minor Warm, Sweet,Slightly Bitter | Lung,Spleen,Heart | supplementing qi and nourishing yin |
|  | Ophiopogon japonicus | methylophiopogonanone  , ophiopogonin | Minor Warm,Sweet,Slightly Bitter | Lung,Spleen,Heart |  |
| Xingding injection | Ginkgo biloba | ginkgo flavonoids, ginkgolide | neutral,Sweet, Bitter | Heart, Lung | invigorating the blood circulation |
| Ciwujia injection | Eleutherococcus senticosus | caffeoylquinic acid, protocatechuic acid, total flavonoids | Pungent, Mild,Bitter | Spleen,Heart,Kidney | invigorating qi |
| Xuesaitong injection | Panax notoginseng | notoginsenoside,  myricetin | Warm,Pungent,Slightly Bitter | Stomach,Liver | invigorating the blood circulation |
| Kudiezi injection | Syzygium aromaticum | total flavonoids,  glochidioboside, | Bitter, Pungent,cold | Heart, Large Intestine | Clearing heat |
| Shuxuening injection | Ginkgo biloba | ginkgo flavonoids, ginkgolid | neutral,Sweet, Bitter | Heart, Lung | invigorating the blood circulation |

***Supplementary table S4*** The list of abbreviations.

| DM | diabetes mellitus |
| --- | --- |
| CHD | coronary heart disease |
| TCM | traditional Chinese medicin |
| TCMIs | traditional Chinese medicine injection |
| RCTs | randomized controlled trials |
| CT | conventional therapies |
| DH | Danhong injection |
| DS | Danshen injection |
| XX | Xixin injection |
| CXQ | Chuanxiongqin injection |
| DSCXQ | Danshenchuanxiongqin injection |
| SM | Shenmai injection |
| CWJ | Ciwujia injection |
| XD | Xingding injection |
| SXN | Shuxuening injection |
| XST | Xuesaitong injection |
| GG | Gegen injection |
| GXN | Guanxinning injection |
| KDS | Kudiezi injection |
| GLP | Gualoupi injection |
| SQ | Shenqi injection |
| SXT | Shuxuening injection |
| FBG | fasting blood glucose |
| PBG | postprandial blood glucose |
| HbA1c | hemoglobinA1c |
| TG | triglycerides |
| TC | total cholesterol |
| HDL | high-density lipoprotein |
| LDL | low-density lipoprotein |
| AEs | adverse events |

***Supplementary table S5*** Bayesian ranking results of network meta-analysis (Total effective rate). The number in each cell represents the posterior probability of the row-defining treatment being ranked at the column-defining position.

| **Treatments** | **Rank of possibility %** | | | | | | | | | | | | | | |
| --- | --- | --- | --- | --- | --- | --- | --- | --- | --- | --- | --- | --- | --- | --- | --- |
|  | 1 | 2 | 3 | 4 | 5 | 6 | 7 | 8 | 9 | 10 | 11 | 12 | 13 | 14 | 15 |
| Danhong  injection | 0 | 0 | 0.2 | 0.6 | 1.8 | 4.2 | 7.5 | 11.4 | 15.2 | 17.2 | 18.6 | 13.9 | 7.6 | 1.8 | 0 |
| Guanxinning  injection | 3.5 | 8.7 | 12.4 | 13.9 | 14.1 | 11.2 | 9.6 | 7.1 | 6.0 | 4.7 | 3.7 | 2.7 | 1.7 | 0.7 | 0 |
| Danshen  injection | 5.7 | 8.4 | 9.8 | 9.8 | 9.4 | 9.0 | 7.7 | 6.4 | 5.7 | 5.8 | 5.8 | 6.2 | 6.2 | 3.9 | 0.3 |
| Chuanxiongqin  injection | 34.6 | 23.7 | 14.3 | 8.9 | 5.6 | 3.8 | 2.8 | 2.0 | 1.4 | 1.1 | 0.8 | 0.6 | 0.4 | 0.1 | 0 |
| Danshen  Chuanxiongqin  injection | 0 | 0.2 | 0.5 | 1.5 | 3.1 | 4.8 | 7.9 | 11.4 | 13.2 | 15.7 | 15.1 | 14.6 | 9.3 | 2.8 | 0 |
| Dengzhanxixin  injection | 0.8 | 3.0 | 5.8 | 8.8 | 10.8 | 12.0 | 11.8 | 11.0 | 9.5 | 8.4 | 7.3 | 5.2 | 4.4 | 1.3 | 0 |
| Gegen  injection | 9.0 | 11.9 | 11.8 | 11.0 | 9.6 | 8.2 | 6.4 | 5.4 | 5.3 | 5.0 | 4.4 | 4.8 | 4.8 | 2.4 | 0.2 |
| Shuxuetong  injection | 0.4 | 1.5 | 3.5 | 5.4 | 7.1 | 8.8 | 9.6 | 9.8 | 10.3 | 9.7 | 9.6 | 11.1 | 9.3 | 4.0 | 0 |
| Shenmai  injection | 0.2 | 1.4 | 3.2 | 5.8 | 8.5 | 11.1 | 11.6 | 12.5 | 11.9 | 10.5 | 9.4 | 7.2 | 5.2 | 1.6 | 0 |
| Kudiezi  injection | 13.5 | 10.8 | 9.3 | 8.4 | 6.7 | 6.0 | 5.1 | 4.4 | 3.9 | 4.1 | 4.8 | 6.1 | 7.8 | 6.9 | 2.3 |
| Ciwujia  injection | 10.3 | 10.4 | 11.2 | 9.7 | 8.8 | 7.0 | 6.4 | 5.4 | 5.1 | 4.8 | 5.0 | 5.8 | 6.0 | 3.5 | 0.6 |
| Xingding  injection | 15.8 | 12.0 | 9.4 | 8.5 | 6.3 | 5.7 | 5.1 | 4.6 | 3.9 | 4.0 | 4.5 | 5.5 | 7.2 | 5.9 | 1.8 |
| Xuesaitong  injection | 0.3 | 0.7 | 1.2 | 1.4 | 2.0 | 2.2 | 3.1 | 3.0 | 3.3 | 4.3 | 5.3 | 8.7 | 16.5 | 31.8 | 16.1 |
| Shuxuening  injection | 6.1 | 7.3 | 7.5 | 6.4 | 6.3 | 6.0 | 5.5 | 5.6 | 5.3 | 4.8 | 5.7 | 7.7 | 12.1 | 10.2 | 3.6 |
| CT | 0 | 0 | 0 | 0 | 0 | 0 | 0 | 0 | 0 | 0 | 0 | 0.1 | 1.8 | 23.1 | 75.1 |

***Supplementary table S6* Bayesian ranking results of network meta-analysis (****EGG effective rate). The number in each cell represents the posterior probability of the row-defining treatment being ranked at the column-defining position.**

| **Treatments** | **Rank of possibility %** | | | | | | | | | | | | |
| --- | --- | --- | --- | --- | --- | --- | --- | --- | --- | --- | --- | --- | --- |
|  | 1 | 2 | 3 | 4 | 5 | 6 | 7 | 8 | 9 | 10 | 11 | 12 | 13 |
| Danhong  injection | 0.1 | 0.9 | 2.8 | 6.8 | 11.7 | 16.2 | 19.4 | 17.7 | 13.5 | 7.3 | 2.7 | 0.7 | 0 |
| Gegen  injection | 1.7 | 4.2 | 6.0 | 8.4 | 9.4 | 10.5 | 11.8 | 12.7 | 12.1 | 11.3 | 7.6 | 3.5 | 0.9 |
| Guanxinning  injection | 8.9 | 21.3 | 24.6 | 19.1 | 12.6 | 7.0 | 3.8 | 1.6 | 0.8 | 0.2 | 0.1 | 0 | 0 |
| Shenqiong  injection | 8.6 | 12.0 | 11.4 | 10.1 | 9.8 | 9.5 | 8.5 | 8.4 | 7.7 | 6.2 | 4.7 | 2.5 | 0.7 |
| Shenmai  injection | 2.3 | 6.6 | 10.3 | 13.9 | 14.2 | 14.3 | 12.6 | 10.2 | 8.2 | 4.7 | 2.1 | 0.6 | 0 |
| Ciwujia  injection | 3.9 | 5.9 | 6.3 | 6.5 | 7.2 | 7.6 | 7.3 | 9.0 | 9.7 | 11.1 | 11.0 | 7.8 | 6.6 |
| Dengzhanxixin  injection | 44.0 | 16.5 | 9.4 | 7.0 | 5.5 | 4.6 | 3.4 | 2.7 | 2.4 | 2.2 | 1.3 | 0.7 | 0.2 |
| Kudiezi  injection | 16.4 | 14.1 | 10.8 | 8.6 | 8.4 | 7.4 | 6.5 | 6.5 | 6.3 | 5.8 | 4.9 | 2.9 | 1.6 |
| Xingding  injection | 5.1 | 7.5 | 6.9 | 7.3 | 8.0 | 8.1 | 8.2 | 8.8 | 9.7 | 11.1 | 11.0 | 7.8 | 6.6 |
| Xuesaitong  injection | 3.5 | 4.4 | 4.8 | 5.2 | 5.0 | 5.2 | 6.8 | 7.5 | 9.2 | 11.1 | 13.0 | 10.7 | 13.6 |
| Danshen  injection | 0.1 | 0.2 | 0.6 | 1.3 | 2.0 | 3.1 | 5.0 | 7.7 | 11.9 | 17.3 | 22.3 | 18.7 | 9.8 |
| Shuxuetong  injection | 5.5 | 6.3 | 6.1 | 5.8 | 6.2 | 6.4 | 6.7 | 7.1 | 8.5 | 10.4 | 11.3 | 9.0 | 10.7 |
| CT | 0 | 0 | 0 | 0 | 0 | 0 | 0 | 0 | 0.2 | 1.7 | 10.2 | 36.3 | 51.6 |

***Supplementary table S7* Bayesian ranking results of network meta-analysis (****Effective rate of angina pectoris). The number in each cell represents the posterior probability of the row-defining treatment being ranked at the column-defining position.**

| **Treatments** | **Rank of possibility %** | | | | | | | |
| --- | --- | --- | --- | --- | --- | --- | --- | --- |
|  | 1 | 2 | 3 | 4 | 5 | 6 | 7 | 8 |
| Danhong  injection | 2.0 | 9.8 | 22.8 | 29.4 | 22.3 | 11.1 | 2.6 | 0 |
| Guanxinning  injection | 28.2 | 31.0 | 18.5 | 11.3 | 6.4 | 3.3 | 1.4 | 0 |
| Shenqiong  injection | 7.3 | 11.4 | 13.0 | 12.8 | 16.2 | 18.3 | 19.6 | 1.4 |
| Ciwujia  injection | 12.0 | 15.0 | 15.6 | 14.7 | 14.3 | 14.9 | 12.9 | 0.6 |
| Shenmai  injection | 44.3 | 21.1 | 13.2 | 8.1 | 6.4 | 4.2 | 2.6 | 0 |
| Danshen  injection | 0.9 | 3.1 | 7.5 | 13.2 | 21.4 | 29.0 | 24.8 | 0.1 |
| Shuxuetong  injection | 5.3 | 8.6 | 9.3 | 10.7 | 13.1 | 19.1 | 29.5 | 4.5 |
| CT | 0 | 0 | 0 | 0 | 0 | 0.1 | 6.5 | 93.4 |

***Supplementary table S8*** Bayesian ranking results of network meta-analysis (FBG). The number in each cell represents the posterior probability of the row-defining treatment being ranked at the column-defining position.

| **Treatments** | **Rank of possibility %** | | | | | | | | |
| --- | --- | --- | --- | --- | --- | --- | --- | --- | --- |
|  | 1 | 2 | 3 | 4 | 5 | 6 | 7 | 8 | 9 |
| Danhong  injection | 0.4 | 2.3 | 8.0 | 18.1 | 26.9 | 26.3 | 13.3 | 4.2 | 0.5 |
| Guanxinning  injection | 3.0 | 3.7 | 4.6 | 6.8 | 9.6 | 13.3 | 22.1 | 21.5 | 15.5 |
| Gualoupi  injection | 1.5 | 1.8 | 2.3 | 3.4 | 5.4 | 7.6 | 14.8 | 24.2 | 38.9 |
| Chuanxiongqin  injection | 19.6 | 15.6 | 12.0 | 10.8 | 8.2 | 6.3 | 4.0 | 1.8 | 0.6 |
| Dengzhanxixin  injection | 38.6 | 17.0 | 12.6 | 10.8 | 8.2 | 6.3 | 4.0 | 1.8 | 0.6 |
| Shenmai  injection | 0.2 | 0.3 | 0.6 | 1.5 | 3.3 | 6.4 | 15.3 | 32.9 | 39.5 |
| Danshen  injection | 6.2 | 9.0 | 11.6 | 16.7 | 18.1 | 17.5 | 13.0 | 6.3 | 1.7 |
| Shuxuetong  injection | 18.0 | 15.6 | 13.6 | 14.8 | 13.1 | 10.7 | 8.6 | 4.1 | 1.4 |
| CT | 12.5 | 34.7 | 34.7 | 14.8 | 3.1 | 0.2 | 0 | 0 | 0 |

***Supplementary table S9*** Bayesian ranking results of network meta-analysis (PBG). The number in each cell represents the posterior probability of the row-defining treatment being ranked at the column-defining position.

| **Treatments** | **Rank of possibility %** | | | | | | |
| --- | --- | --- | --- | --- | --- | --- | --- |
|  | 1 | 2 | 3 | 4 | 5 | 6 | 7 |
| Danhong  injection | 2.0 | 7.7 | 18.9 | 29.9 | 28.2 | 12.2 | 1.2 |
| Gualoupi  injection | 22.0 | 16.5 | 12.4 | 14.0 | 15.8 | 14.3 | 5.0 |
| Chuanxiongqin  injection | 6.6 | 8.0 | 9.7 | 13.2 | 20.2 | 29.9 | 12.4 |
| Dengzhanxixin  injection | 24.3 | 18.3 | 13.8 | 13.9 | 14.0 | 12.0 | 3.7 |
| Shenmai  injection | 0.7 | 1.2 | 1.7 | 3.1 | 6.4 | 16.9 | 70.1 |
| Shuxuetong  injection | 30.8 | 13.8 | 9.5 | 11.1 | 12.6 | 14.6 | 7.7 |
| CT | 13.7 | 34.5 | 34.1 | 14.8 | 2.7 | 0.2 | 0 |

***Supplementary table S10* Bayesian ranking results of network meta-analysis (HbA1c). The number in each cell represents the posterior probability of the row-defining treatment being ranked at the column-defining position.**

| **Treatments** | **Rank of possibility %** | | | | | |
| --- | --- | --- | --- | --- | --- | --- |
|  | 1 | 2 | 3 | 4 | 5 | 6 |
| Danhong  injection | 0.1 | 0.6 | 5.5 | 24.4 | 57.6 | 11.8 |
| Guanxinning  injection | 0 | 0.2 | 0.9 | 4.2 | 13.2 | 81.6 |
| Gualoupi  injection | 56.1 | 20.2 | 11.8 | 8.4 | 2.9 | 0.6 |
| Chuanxiongqin  injection | 9.9 | 15.7 | 20.3 | 32.7 | 17.2 | 4.3 |
| Shuxuetong  injection | 25.2 | 25.3 | 20.1 | 18.7 | 9.0 | 1.7 |
| CT | 8.7 | 38.0 | 41.5 | 11.7 | 0.1 | 0 |

***Supplementary table S11* Bayesian ranking results of network meta-analysis (TC). The number in each cell represents the posterior probability of the row-defining treatment being ranked at the column-defining position.**

| **Treatments** | **Rank of possibility %** | | | | | | | | | | |
| --- | --- | --- | --- | --- | --- | --- | --- | --- | --- | --- | --- |
|  | 1 | 2 | 3 | 4 | 5 | 6 | 7 | 8 | 9 | 10 | 11 |
| Danhong  injection | 0 | 0.1 | 0.6 | 3.0 | 9.8 | 24.9 | 44.0 | 15.4 | 2.2 | 0 | 0 |
| Chuanxiongqin  injection | 18.9 | 18.8 | 14.8 | 14.7 | 13.8 | 10.7 | 5.6 | 2.2 | 0.6 | 0 | 0 |
| Danshen Chuanxiongqin  injection | 0 | 0 | 0 | 0 | 0.2 | 0.8 | 4.8 | 29.3 | 62.4 | 2.4 | 0.1 |
| Dengzhanxixin  injection | 0 | 0 | 0 | 0 | 0 | 0 | 0 | 0.1 | 0.9 | 43.4 | 55.5 |
| Gegen  injection | 0 | 0.1 | 0.2 | 0.8 | 2.6 | 6.7 | 17.3 | 42.6 | 28.0 | 1.6 | 0.1 |
| Shenmai  injection | 9.5 | 12.0 | 12.3 | 14.2 | 17.3 | 16.4 | 10.9 | 5.0 | 2.2 | 0.2 | 0 |
| Danshen  injection | 0 | 0 | 0 | 0 | 0 | 0 | 0.1 | 0.6 | 2.6 | 52.3 | 44.4 |
| Shuxuetong  injection | 1.7 | 5.4 | 9.8 | 17.6 | 27.4 | 25.7 | 10.2 | 2.2 | 0.2 | 0 | 0 |
| Kudiezi  injection | 33.0 | 20.9 | 12.3 | 11.2 | 10.4 | 7.0 | 3.5 | 1.2 | 0.4 | 0 | 0 |
| Xingding  injection | 32.4 | 21.2 | 13.1 | 11.1 | 9.8 | 7.0 | 3.6 | 1.5 | 0.4 | 0 | 0 |
| CT | 4.6 | 21.5 | 36.8 | 27.4 | 8.9 | 0.8 | 0 | 0 | 0 | 0 | 0 |

***Supplementary table S12* Bayesian ranking results of network meta-analysis (TG). The number in each cell represents the posterior probability of the row-defining treatment being ranked at the column-defining position.**

| **Treatments** | **Rank of possibility %** | | | | | | | | | | | |
| --- | --- | --- | --- | --- | --- | --- | --- | --- | --- | --- | --- | --- |
|  | 1 | 2 | 3 | 4 | 5 | 6 | 7 | 8 | 9 | 10 | 11 | 12 |
| Danhong  injection | 2.7 | 4.7 | 7.1 | 11.5 | 14.5 | 16.3 | 15.0 | 12.5 | 8.4 | 5.1 | 2.2 | 0 |
| Chuanxiongqin  injection | 4.5 | 4.7 | 4.8 | 5.4 | 7.2 | 9.3 | 9.7 | 10.7 | 11.6 | 13.2 | 19.1 | 0 |
| Danshen Chuanxiongqin  injection | 0 | 0.1 | 0.4 | 1.3 | 3.0 | 6.5 | 11.4 | 17.0 | 22.6 | 23.5 | 14.2 | 0 |
| Dengzhanxixin  injection | 0.1 | 0.3 | 0.6 | 1.2 | 2.5 | 4.3 | 6.9 | 10.8 | 15.5 | 23.3 | 34.4 | 0 |
| Gegen  injection | 1.8 | 3.5 | 4.9 | 7.2 | 11.0 | 13.8 | 14.9 | 14.6 | 12.8 | 9.9 | 5.6 | 0 |
| Shenmai  injection | 15.6 | 12.2 | 10.5 | 9.5 | 10.4 | 10.3 | 8.5 | 7.5 | 6.2 | 5.0 | 4.2 | 0 |
| Danshen  injection | 0 | 0 | 0 | 0 | 0 | 0 | 0 | 0 | 0 | 0 | 0 | 100 |
| Shuxuetong  injection | 14.7 | 16.1 | 13.9 | 13.2 | 13.2 | 10.7 | 8.0 | 5.2 | 2.9 | 1.5 | 0.6 | 0 |
| Kudiezi  injection | 18.9 | 12.5 | 9.0 | 9.6 | 9.5 | 9.0 | 8.3 | 6.8 | 5.9 | 5.8 | 4.8 | 0 |
| Xingding  injection | 19.7 | 13.8 | 9.6 | 9.2 | 9.6 | 8.2 | 7.9 | 6.6 | 5.9 | 4.8 | 4.8 | 0 |
| Gualoupi  injection | 15.4 | 10.2 | 7.1 | 7.3 | 8.0 | 8.6 | 8.8 | 8.3 | 8.3 | 8.0 | 10.0 | 0 |
| CT | 6.5 | 21.9 | 32.3 | 24.6 | 11.1 | 3.0 | 0.5 | 0 | 0 | 0 | 0 | 0 |

***Supplementary table S13* Bayesian ranking results of network meta-analysis (HDL). The number in each cell represents the posterior probability of the row-defining treatment being ranked at the column-defining position.**

| **Treatments** | **Rank of possibility %** | | | | | | | | | | |
| --- | --- | --- | --- | --- | --- | --- | --- | --- | --- | --- | --- |
|  | 1 | 2 | 3 | 4 | 5 | 6 | 7 | 8 | 9 | 10 | 11 |
| Danhong  injection | 0.1 | 0.4 | 1.5 | 5.4 | 10.6 | 15.9 | 18.3 | 16.6 | 13.8 | 11.6 | 5.9 |
| Chuanxiongqin  injection | 54.1 | 18.5 | 11.8 | 6.7 | 4.1 | 2.2 | 1.2 | 0.7 | 0.4 | 0.3 | 0.1 |
| Danshen Chuanxiongqin  injection | 16.7 | 30.5 | 27.5 | 15.0 | 6.4 | 2.6 | 1.0 | 0.3 | 0.1 | 0 | 0 |
| Dengzhanxixin  injection | 0.5 | 2.0 | 4.0 | 8.1 | 12.5 | 14.9 | 15.2 | 12.5 | 11.1 | 12.0 | 7.1 |
| Gegen  injection | 18.7 | 28.3 | 23.6 | 14.9 | 7.6 | 4.0 | 1.9 | 0.7 | 0.3 | 0.1 | 0 |
| Shenmai  injection | 0.5 | 1.6 | 3.8 | 6.3 | 10.4 | 12.1 | 13.1 | 12.0 | 11.3 | 15.8 | 13.1 |
| Shuxuetong  injection | 1.7 | 4.5 | 9.1 | 15.8 | 17.5 | 16.6 | 12.6 | 8.7 | 6.0 | 5.0 | 2.4 |
| Kudiezi  injection | 3.9 | 7.4 | 9.0 | 13.3 | 14.4 | 12.5 | 10.6 | 8.1 | 6.7 | 8.6 | 5.6 |
| Xingding  injection | 3.7 | 6.6 | 9.2 | 13.0 | 13.5 | 13.5 | 11.2 | 8.5 | 7.3 | 8.0 | 5.4 |
| Gualoupi  injection | 0.1 | 0.3 | 0.5 | 1.5 | 2.7 | 3.7 | 5.4 | 6.8 | 7.8 | 15.7 | 55.4 |
| CT | 0 | 0 | 0 | 0 | 0.3 | 2.0 | 9.5 | 25.1 | 35.3 | 23.0 | 4.8 |

***Supplementary table S14*** Bayesian ranking results of network meta-analysis (LDL). The number in each cell represents the posterior probability of the row-defining treatment being ranked at the column-defining position.

| **Treatments** | **Rank of possibility %** | | | | | | | | | | |
| --- | --- | --- | --- | --- | --- | --- | --- | --- | --- | --- | --- |
|  | 1 | 2 | 3 | 4 | 5 | 6 | 7 | 8 | 9 | 10 | 11 |
| Danhong  injection | 0.2 | 1.0 | 7.8 | 27.1 | 32.4 | 21.4 | 8.2 | 1.8 | 0.2 | 0 | 0 |
| Chuanxiongqin  injection | 0.7 | 2.3 | 4.0 | 8.3 | 10.2 | 14.4 | 20.1 | 18.7 | 13.0 | 6.8 | 1.6 |
| Danshen Chuanxiongqin  injection | 0 | 0 | 0 | 0 | 0 | 0 | 0 | 0.6 | 4.2 | 36.3 | 58.9 |
| Dengzhanxixin  injection | 0 | 0 | 0 | 0 | 0.2 | 1.4 | 6.5 | 21.6 | 46.4 | 22.0 | 1.9 |
| Gegen  injection | 0.7 | 2.9 | 9.3 | 23.6 | 26.5 | 20.8 | 11.2 | 4.2 | 0.7 | 0.1 | 0 |
| Shenmai  injection | 0.2 | 0.3 | 0.7 | 1.5 | 2.2 | 3.3 | 5.8 | 8.6 | 13.0 | 27.8 | 36.6 |
| Shuxuetong  injection | 0 | 0 | 0.4 | 2.4 | 6.5 | 16.4 | 28.9 | 29.5 | 12.9 | 3.0 | 0.1 |
| Kudiezi  injection | 45.0 | 25.8 | 12.1 | 7.5 | 4.4 | 2.9 | 1.4 | 0.6 | 0.2 | 0 | 0 |
| Xingding  injection | 43.2 | 27.5 | 12.6 | 7.7 | 4.3 | 2.7 | 1.4 | 0.5 | 0.1 | 0 | 0 |
| Gualoupi  injection | 2.4 | 4.2 | 6.7 | 12.4 | 12.8 | 16.7 | 16.6 | 13.9 | 9.4 | 4.0 | 0.9 |
| CT | 7.5 | 35.9 | 46.5 | 9.4 | 0 | 0 | 0 | 0 | 0 | 0 | 0 |

***Supplementary table S15* Bayesian ranking results of network meta-analysis (Frequency of angina pectoris). The number in each cell represents the posterior probability of the row-defining treatment being ranked at the column-defining position.**

| **Treatments** | **Rank of possibility %** | | | | | |
| --- | --- | --- | --- | --- | --- | --- |
|  | 1 | 2 | 3 | 4 | 5 | 6 |
| Danhong  injection | 0 | 0 | 0.3 | 3.3 | 21.6 | 74.7 |
| Guanxinning  injection | 0 | 3.1 | 22.8 | 41.4 | 29.1 | 3.6 |
| Gualoupi  injection | 0 | 6.5 | 21.9 | 25.9 | 30.6 | 15.1 |
| Chuanxiongqin  injection | 0.3 | 17.1 | 36.9 | 23.1 | 16.5 | 6.1 |
| Shuxuetong  injection | 7.7 | 65.4 | 18.1 | 6.2 | 2.2 | 0.4 |
| CT | 92.0 | 8.0 | 0 | 0 | 0 | 0 |

***Supplementary table S16* Bayesian ranking results of network meta-analysis (Duration of angina pectoris). The number in each cell represents the posterior probability of the row-defining treatment being ranked at the column-defining position.**

| **Treatments** | **Rank of possibility %** | | | | | |
| --- | --- | --- | --- | --- | --- | --- |
|  | 1 | 2 | 3 | 4 | 5 | 6 |
| Danhong  injection | 0 | 3.0 | 24 | 42.9 | 21.6 | 74.7 |
| Danshen  injection | 0 | 5.8 | 24.7 | 34.4 | 34.5 | 0.7 |
| Danshenchuanxiongqin  injection | 0 | 68.6 | 20.7 | 6.3 | 4.3 | 0.1 |
| Shenmai  injection | 0 | 22.6 | 30.4 | 16.1 | 28.3 | 2.6 |
| Shuxuetong  injection | 0 | 0 | 0.1 | 0.4 | 3.2 | 96.3 |
| CT | 100 | 0 | 0 | 0 | 0 | 0 |

**
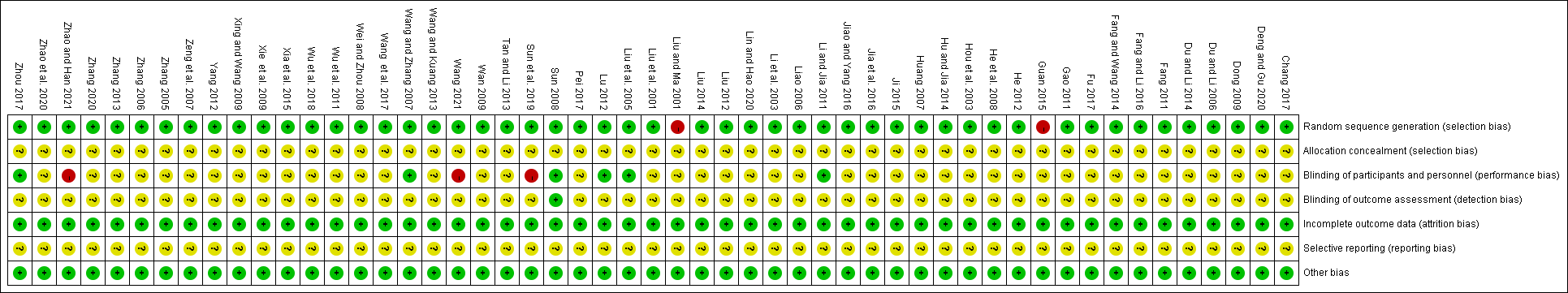
**

***Supplementary figure S1*** Summary of results from assessment of studies using the Cochrane risk of bias tool.

**
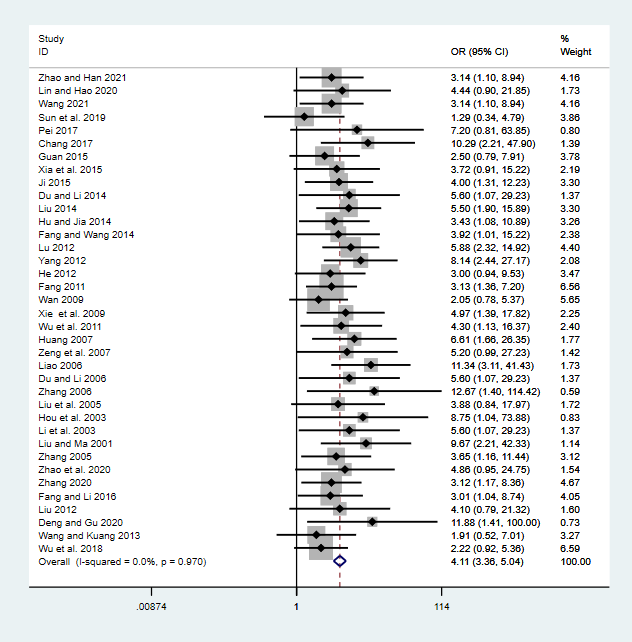
**

***Supplementary figure S2*** The Forest plots of total effective rate.


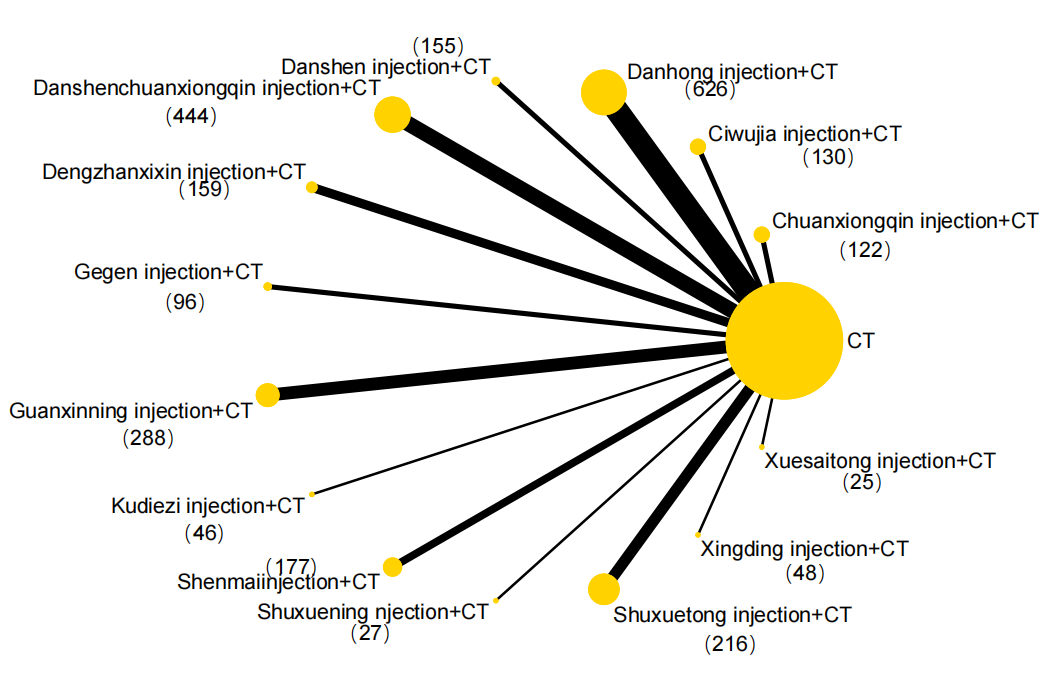

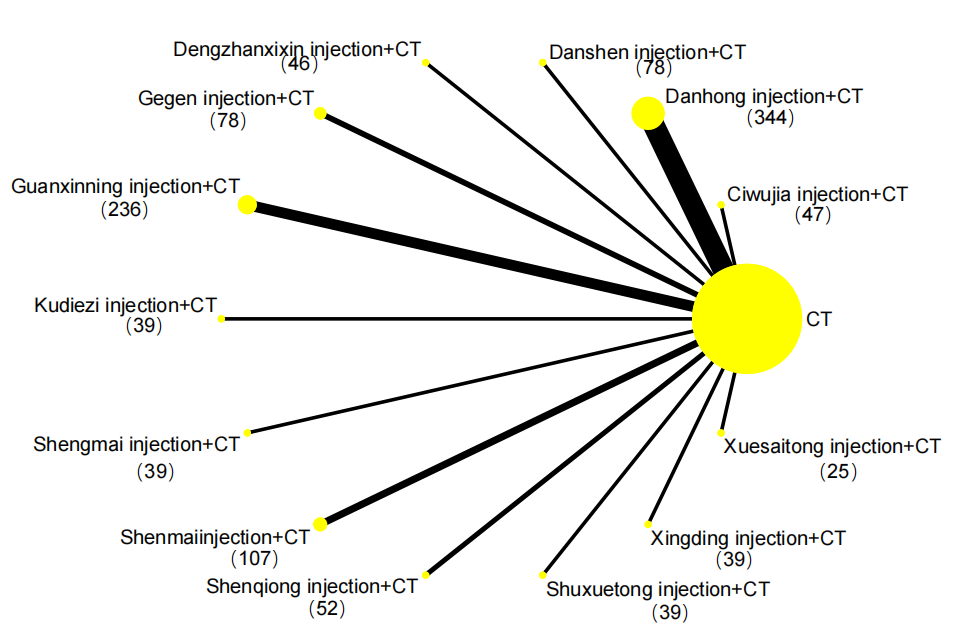


A B


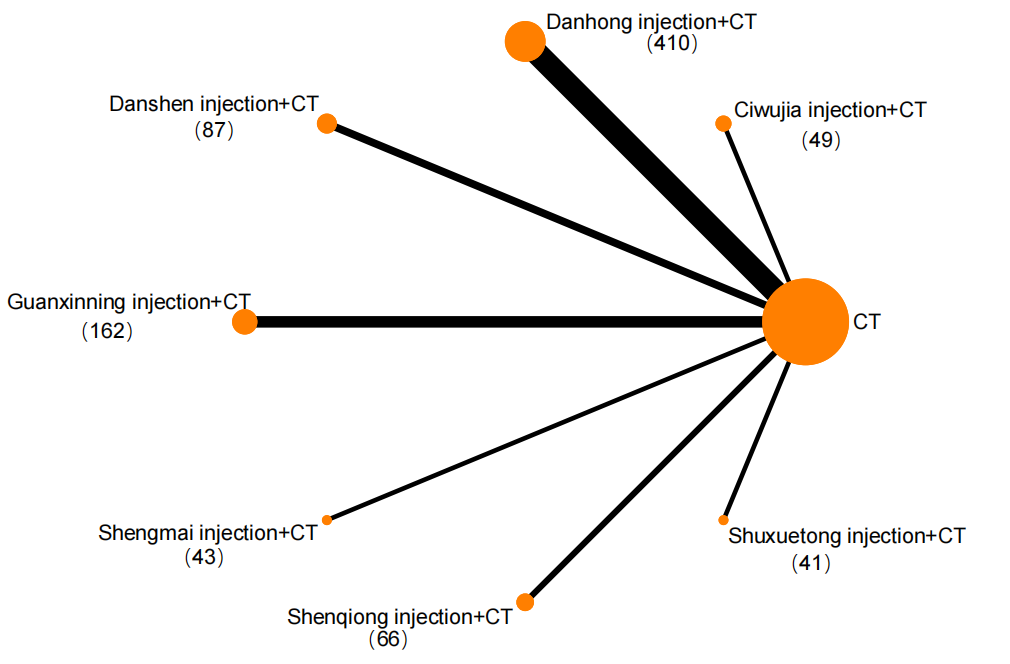

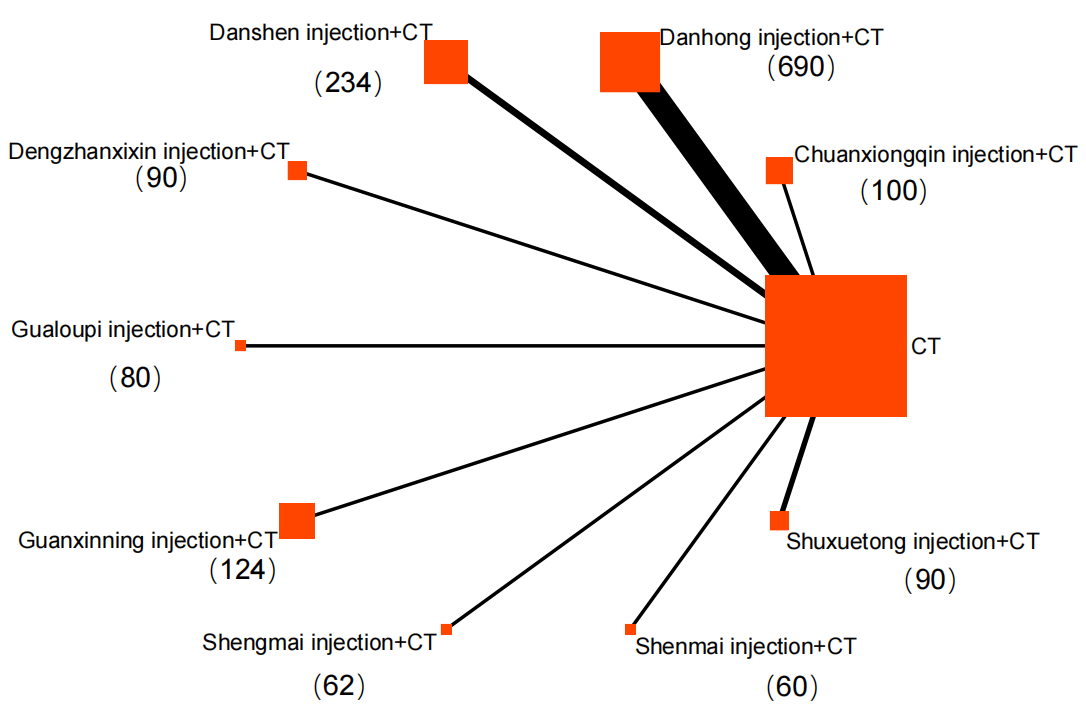


**C D**

**
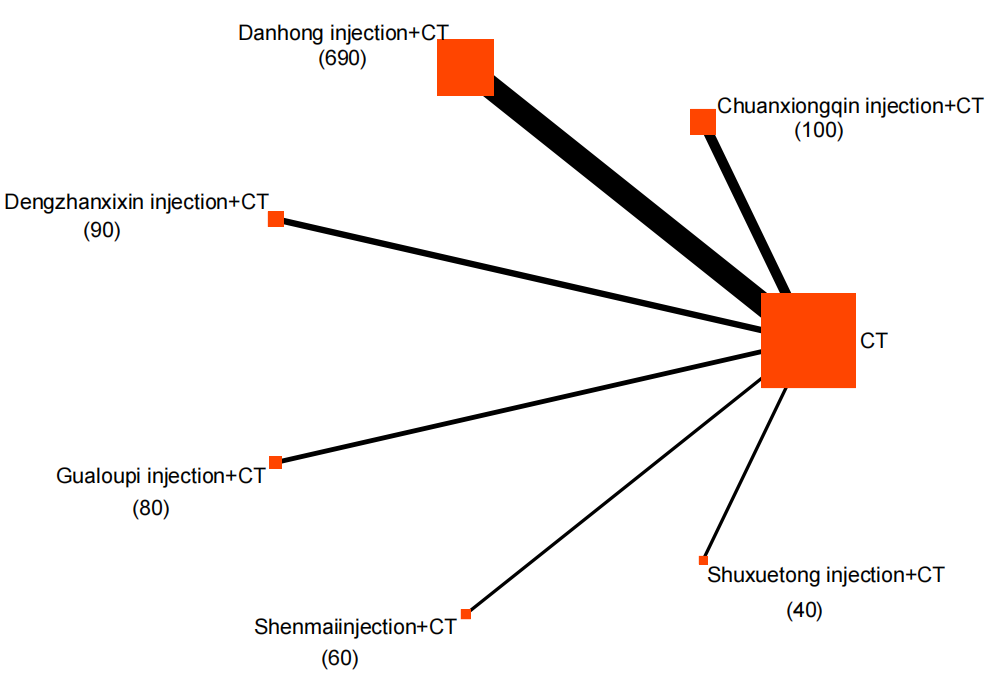
** **
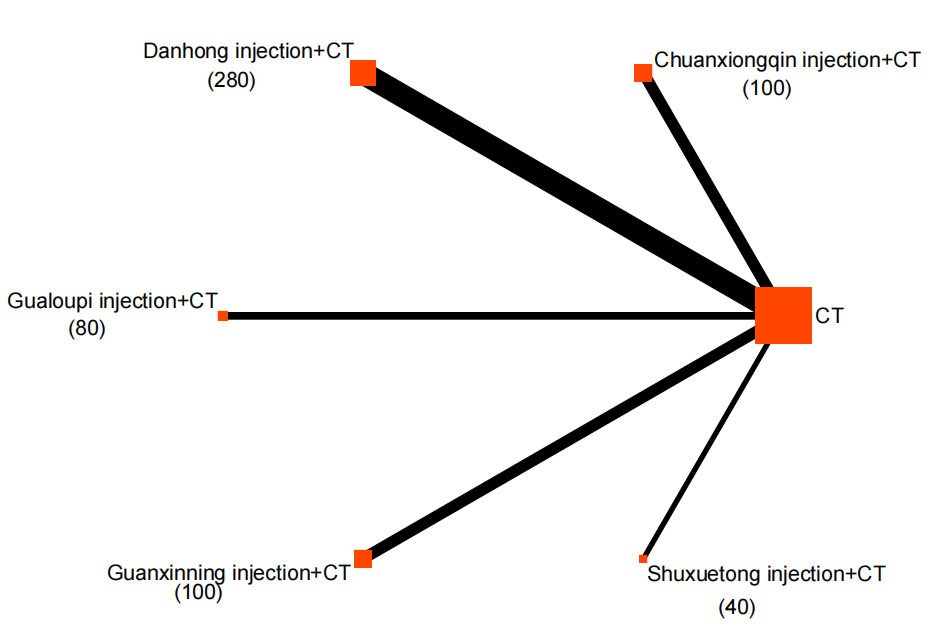
**

**E F**

**
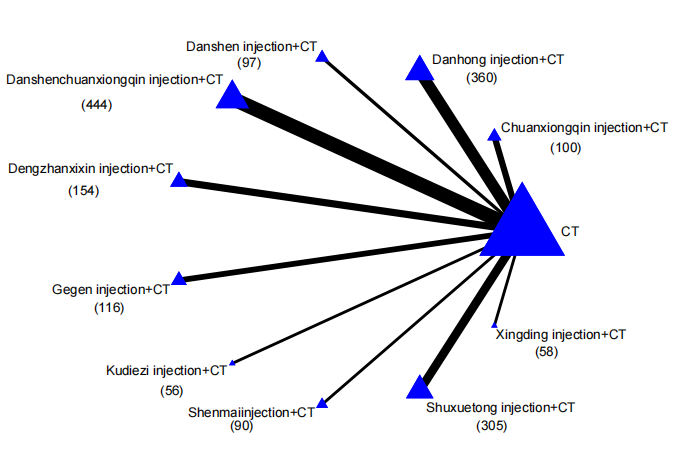
** **
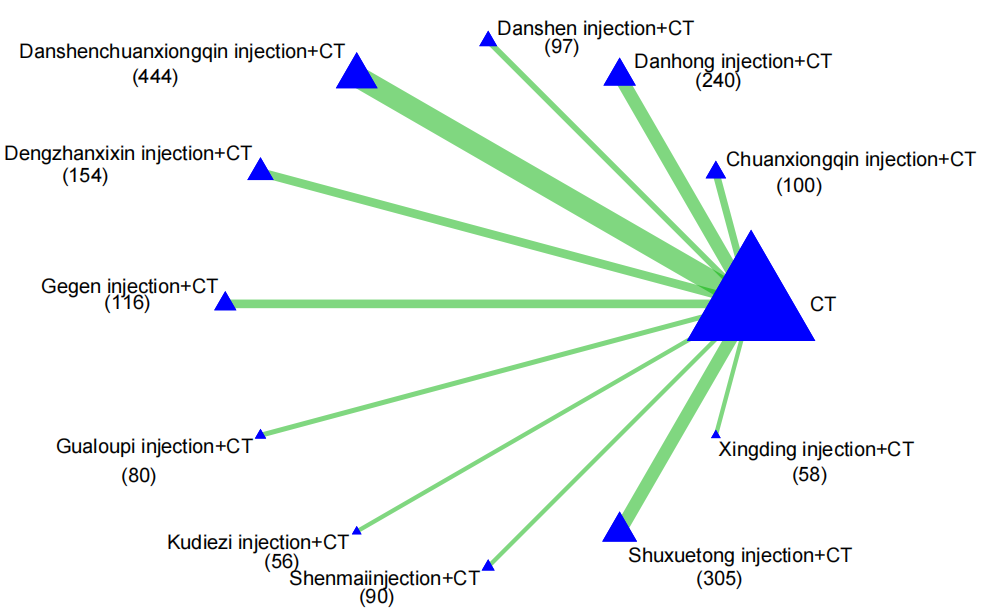
**

**G H**

**
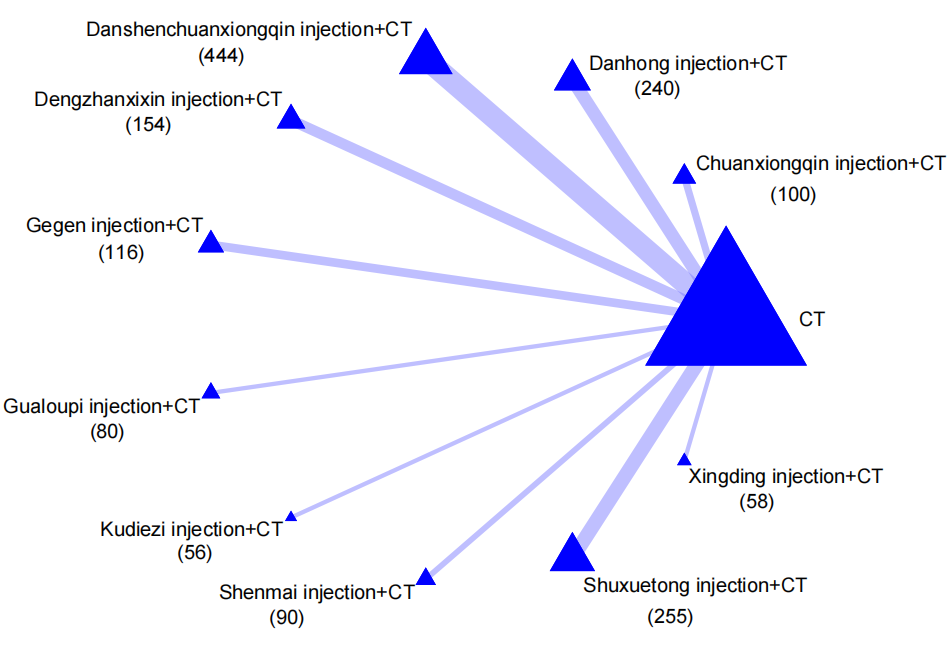
** **
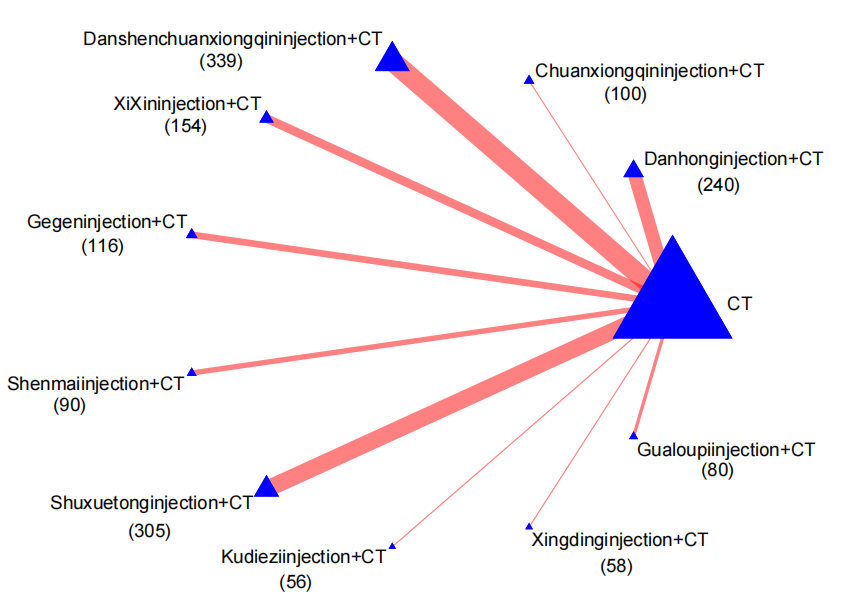
**

**I J**

**
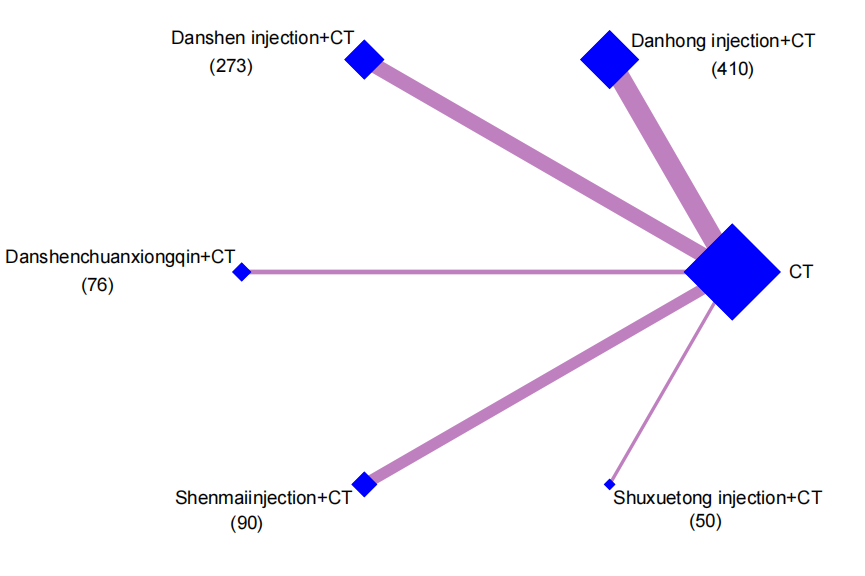
** **
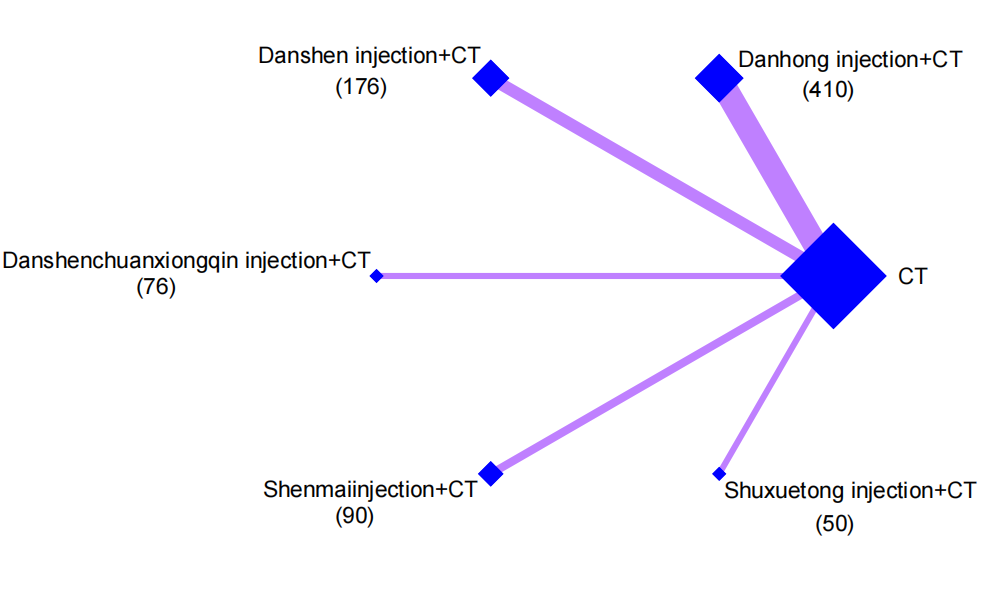
**

**K L**

***Supplementary figure S3*** Network graphs of comparisons on different outcomes of treatments in different groups of patients with DM-CHD. **(A)** total effective rate; **(B)** EGG effective rate; **(C)** Effective rate of angina pectoris; **(D)** FBG; **(E)** PBG; **(F)** HbA1c; **(G)** TC; **(H)** TG; **(I)** HDL; **(J)** LDL; **(K)** Frequency of angina pectoris; **(L)** Duration of angina pectoris.


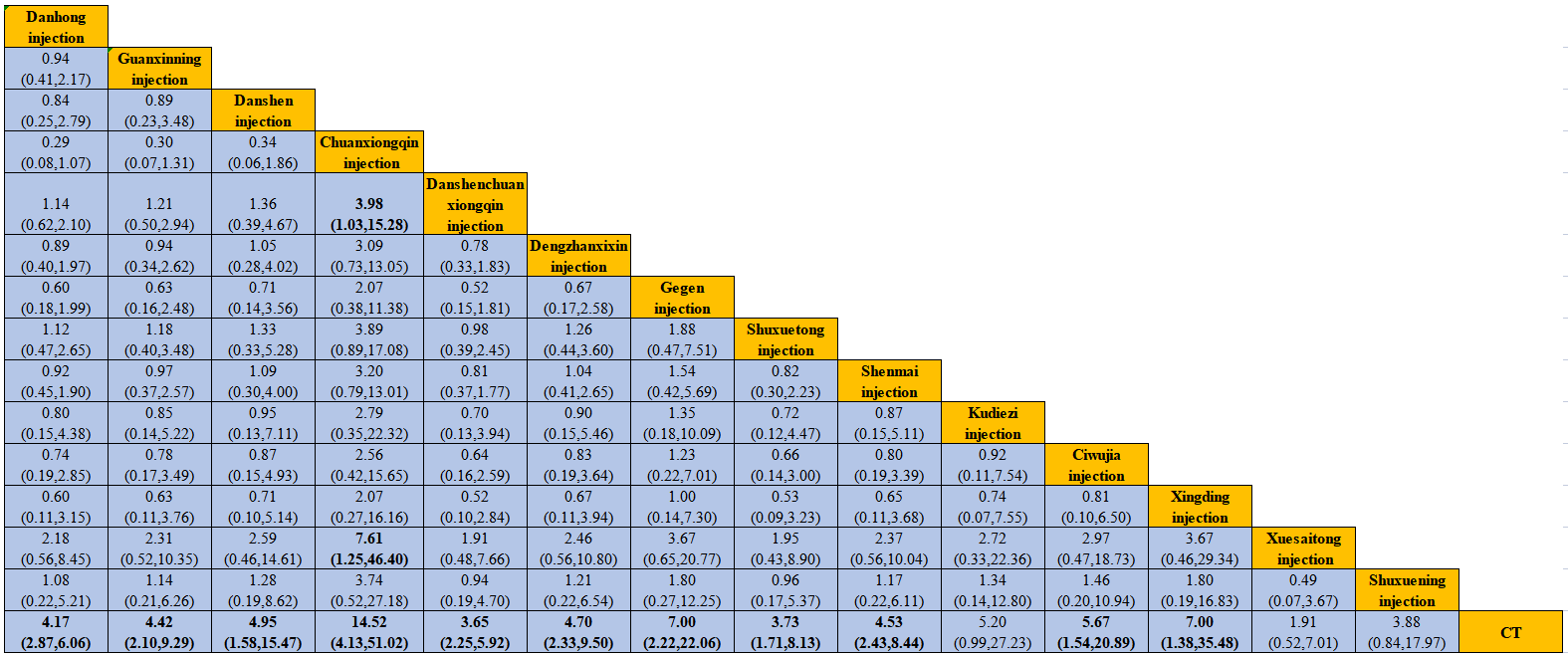


**A**

**
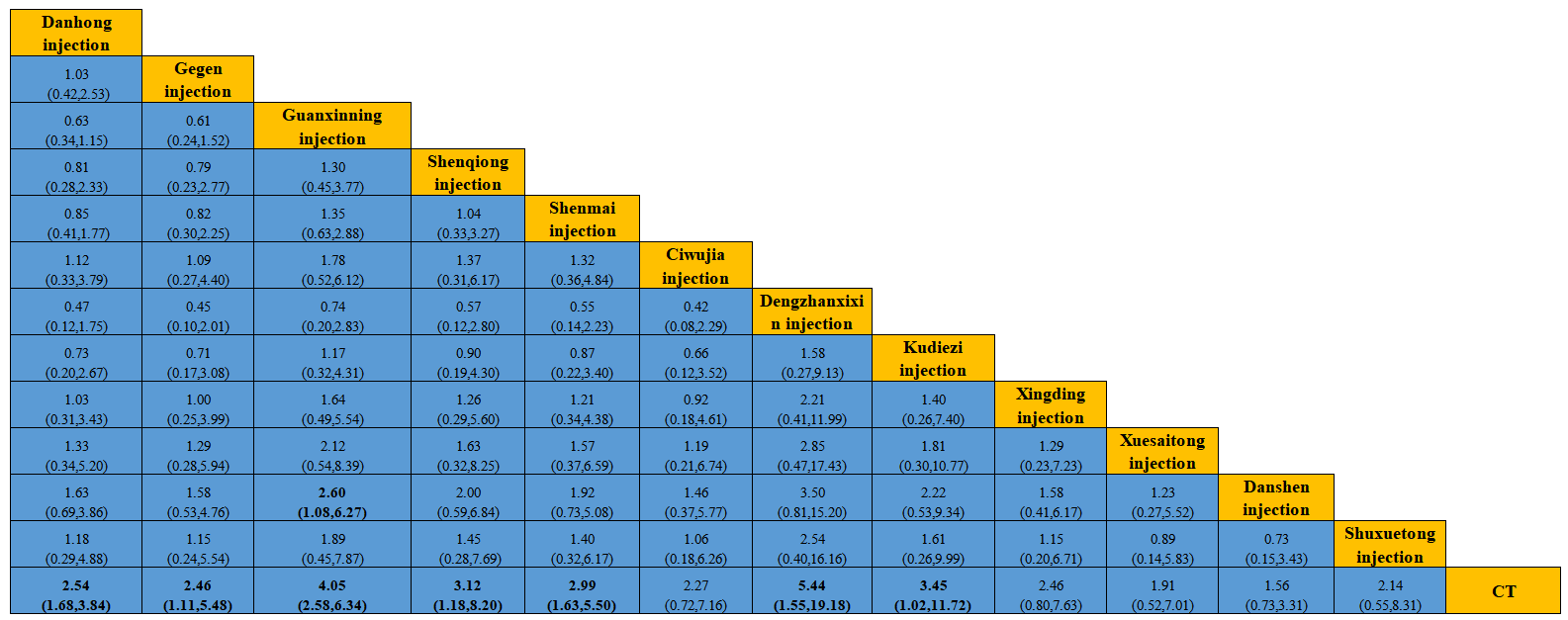
**

**B**

**
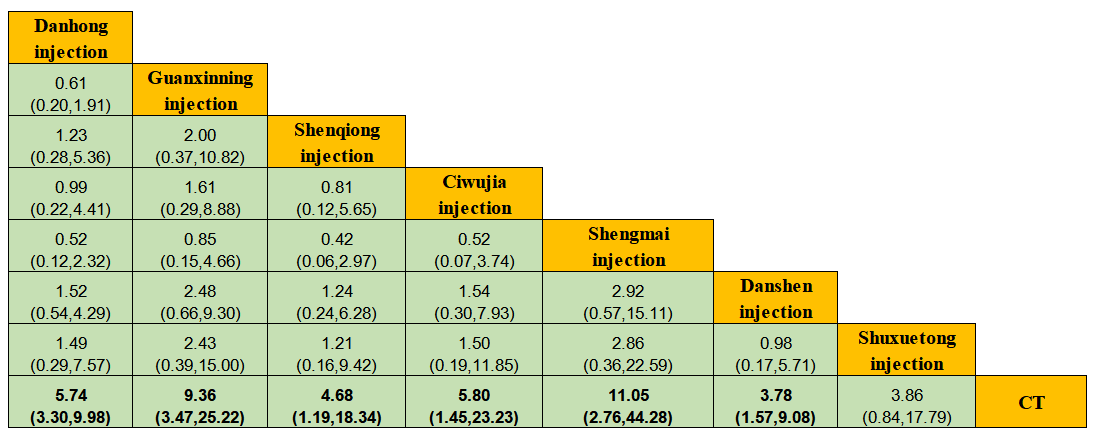
**

**C**

**
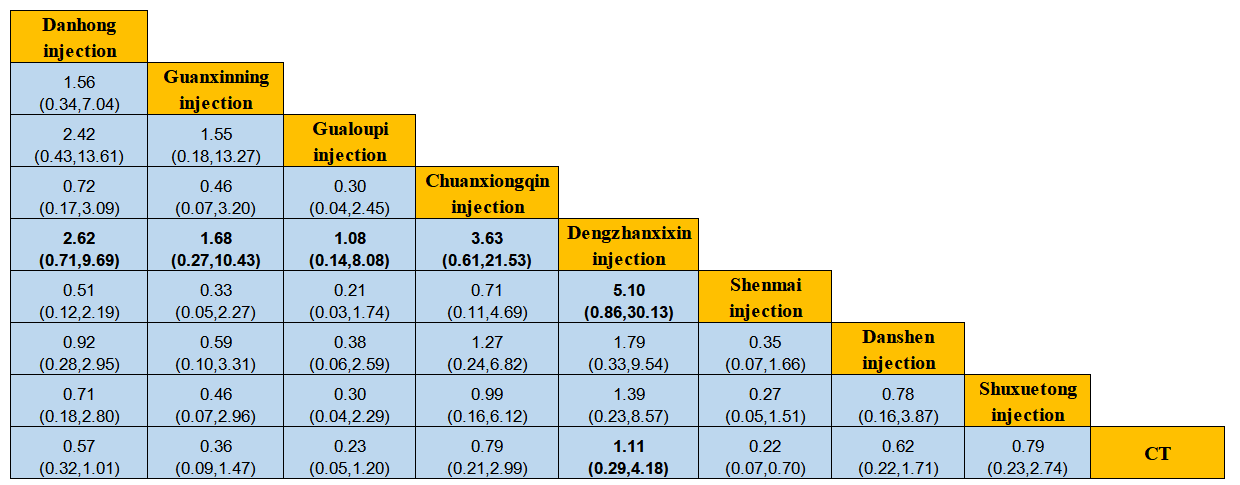
**

**D**

**
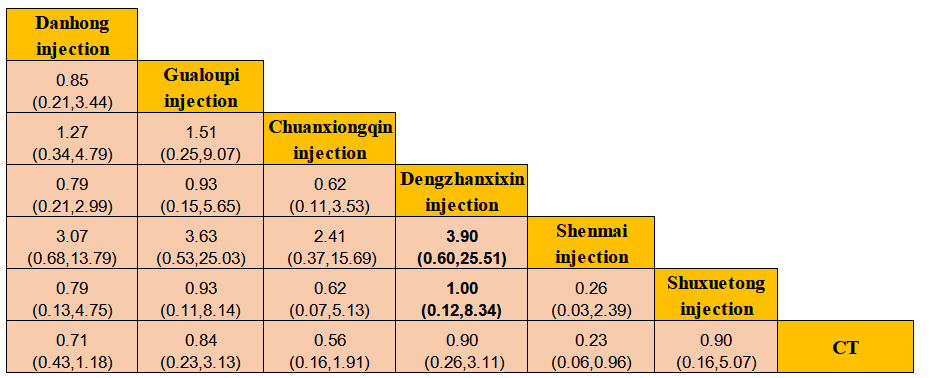
**
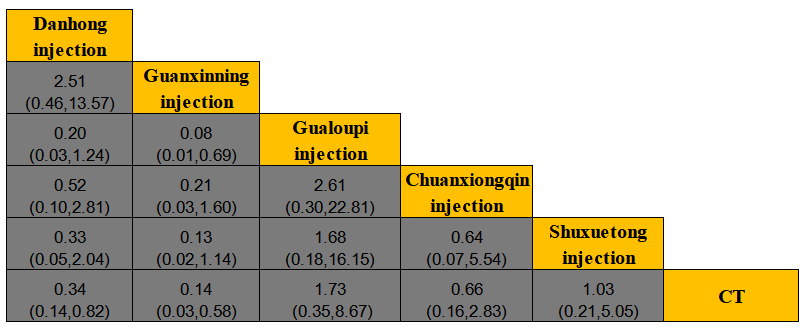


**E F**

**
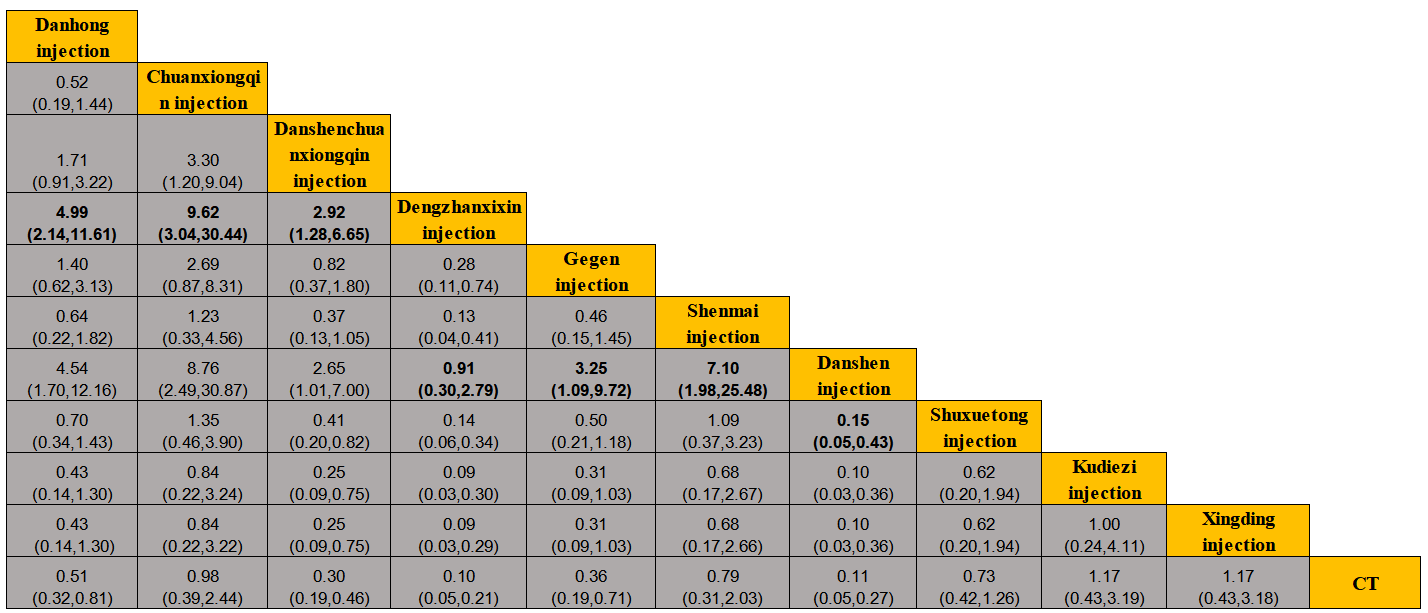
**

**G**

**
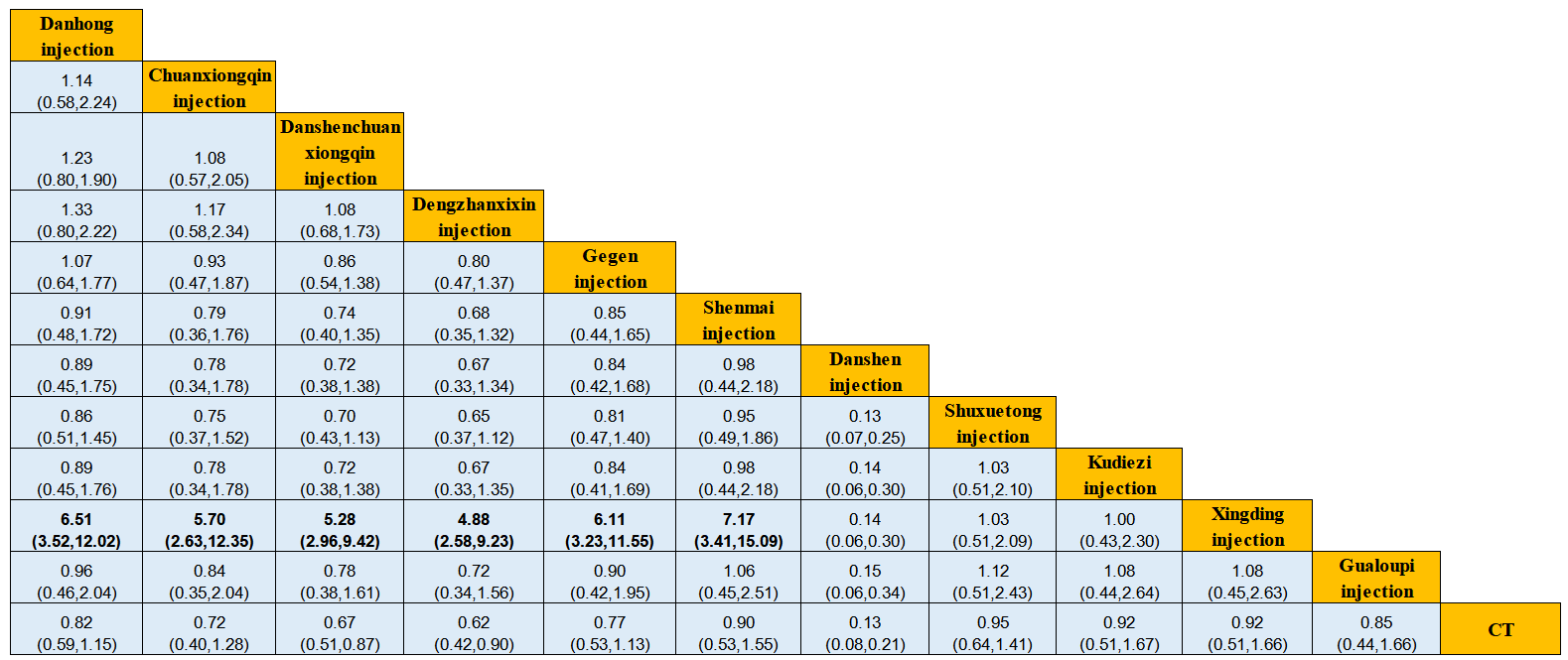
**

**H**

**
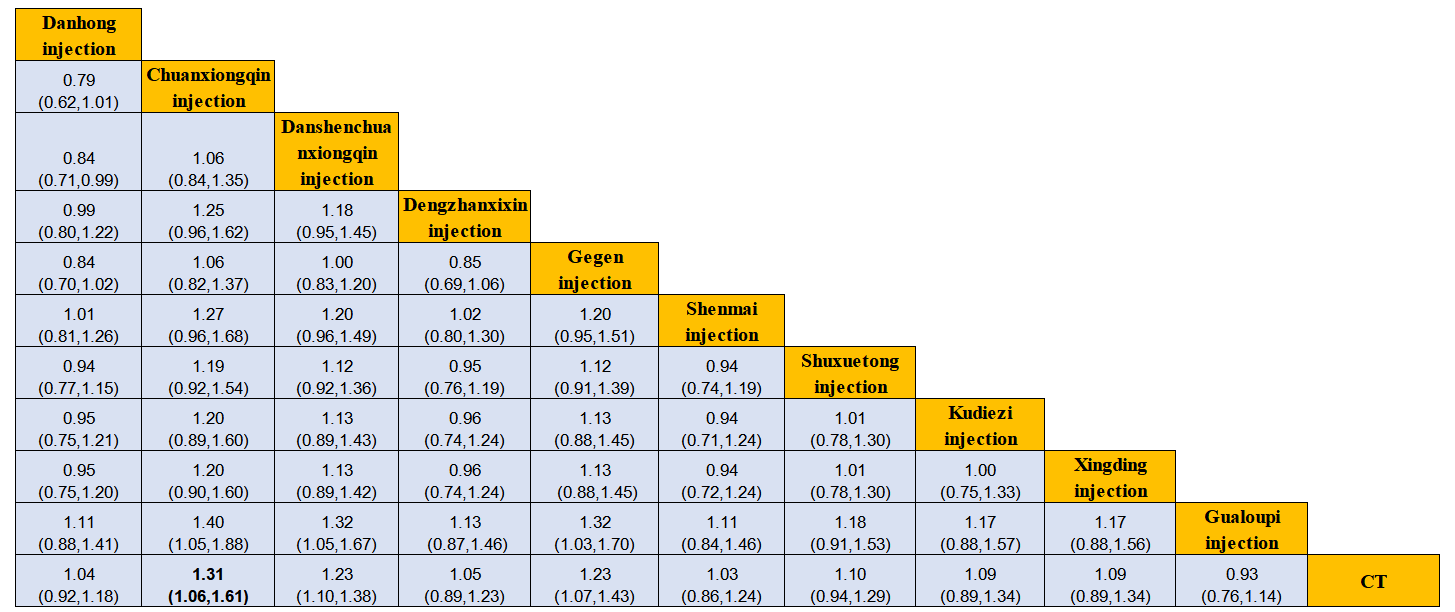
**

**I**


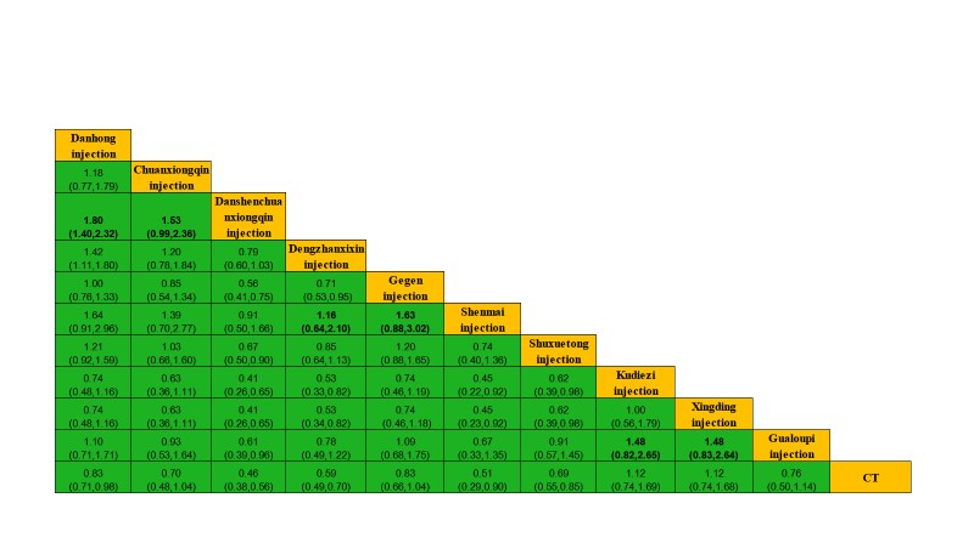


**J**

**
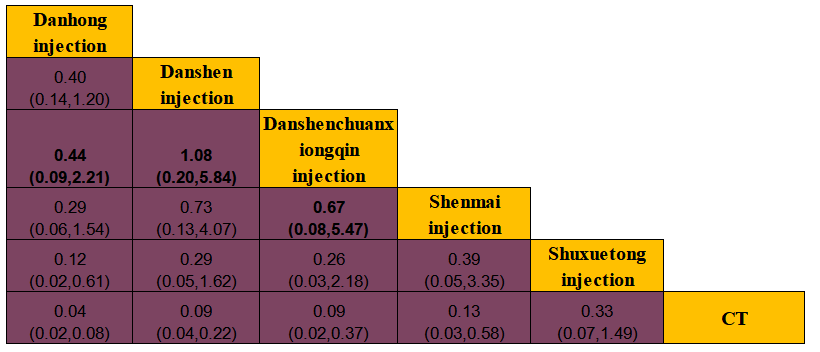
**

**K**

**
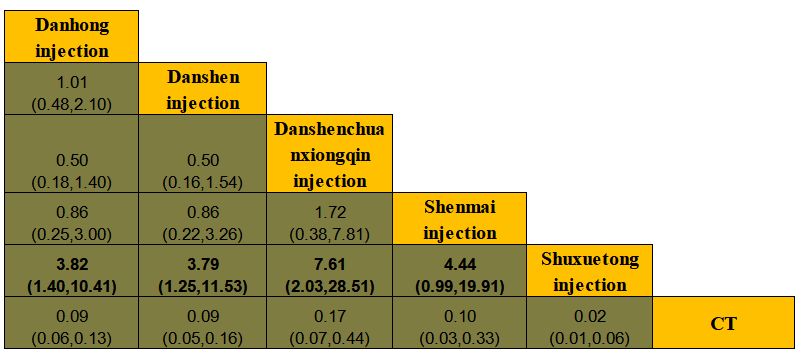
**

**L**

***Supplementary figure S4*** Final results of the systematic meta−analysis. **(A)** total effective rate; **(B)** EGG effective rate; **(C)** Effective rate of angina pectoris; **(D)** FBG; **(E)** PBG; **(F)** HbA1c; **(G)** TC; **(H)** TG; **(I)** HDL; **(J)** LDL; **(K)** Frequency of angina pectoris; **(L)** Duration of angina pectoris.

**
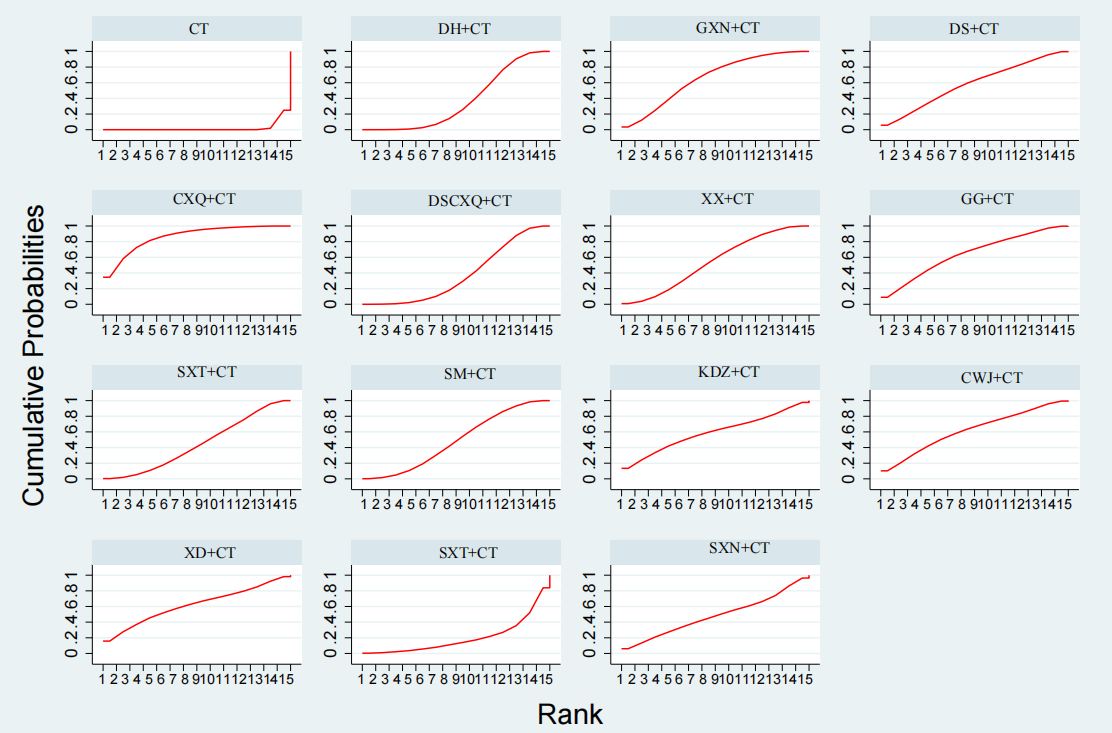
** **
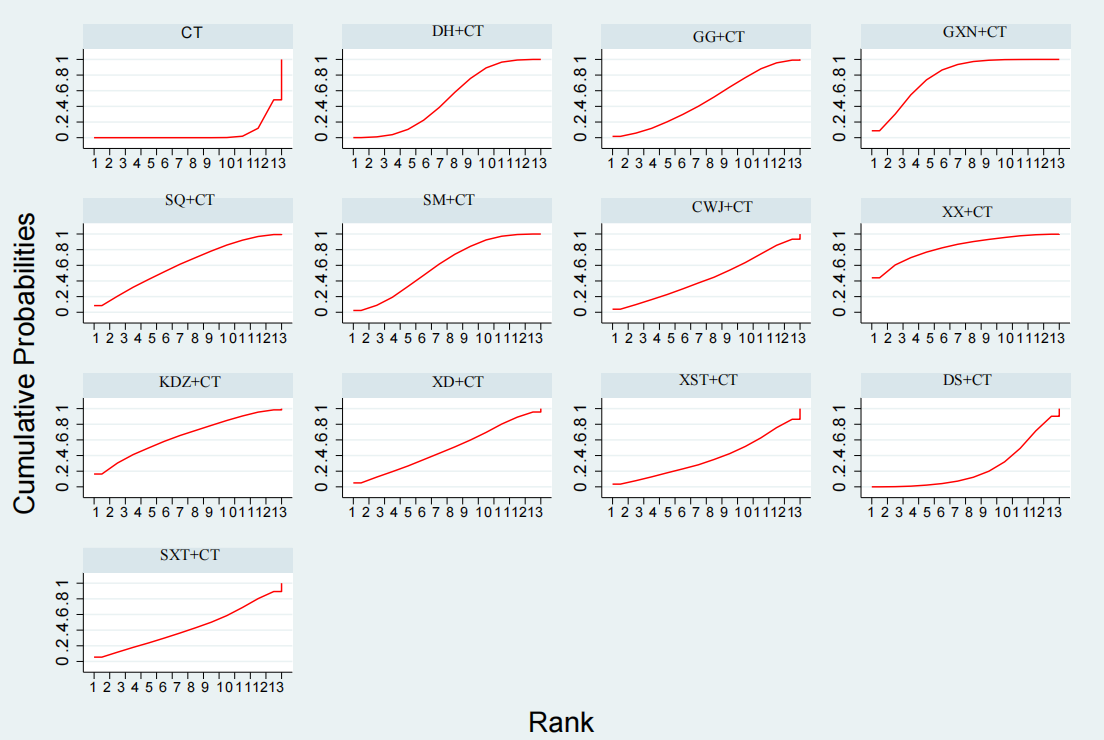
**

**A B**

**
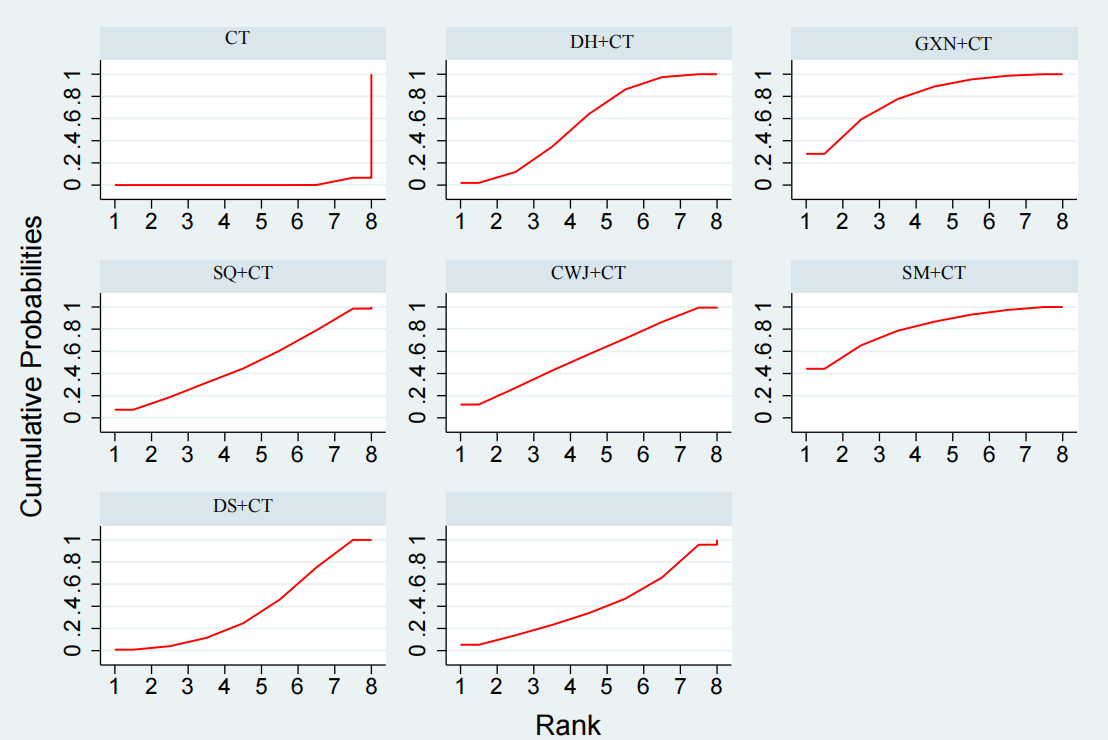
** **
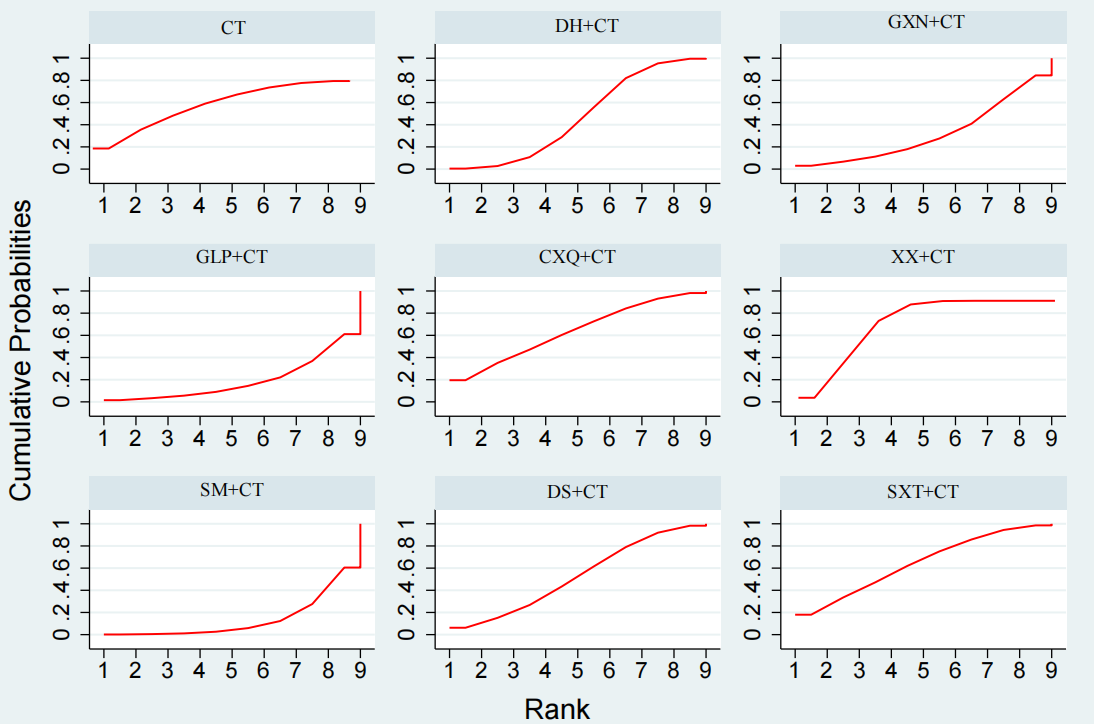
**

**C D**

**
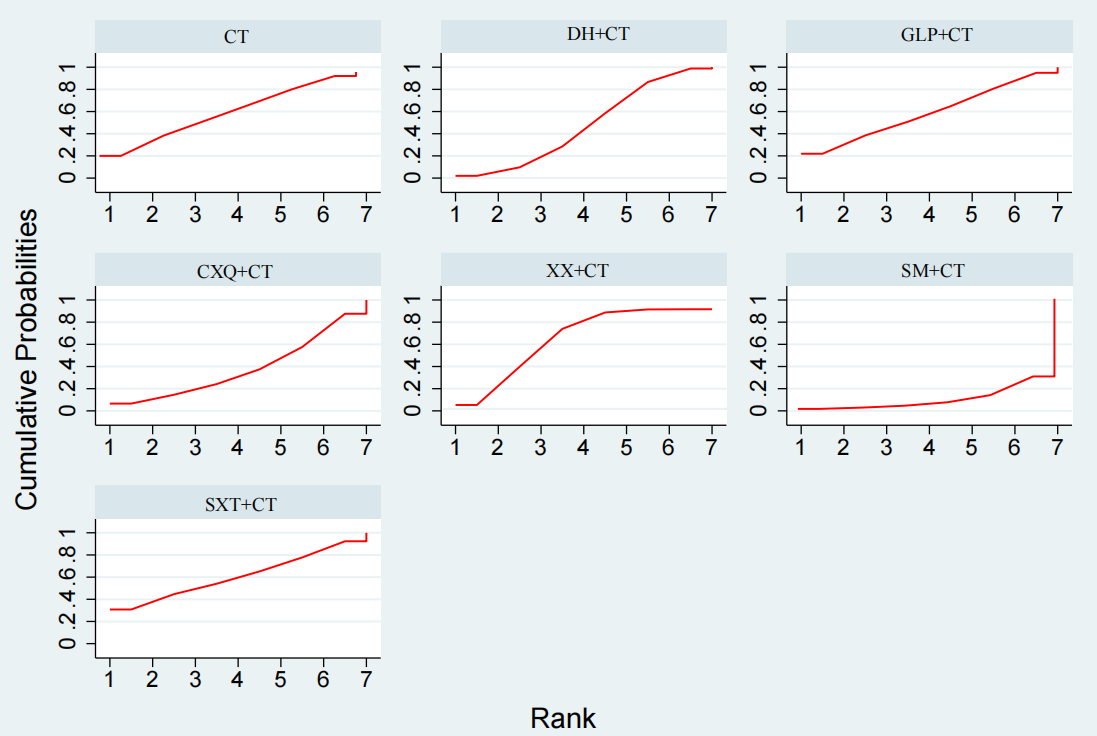
** **
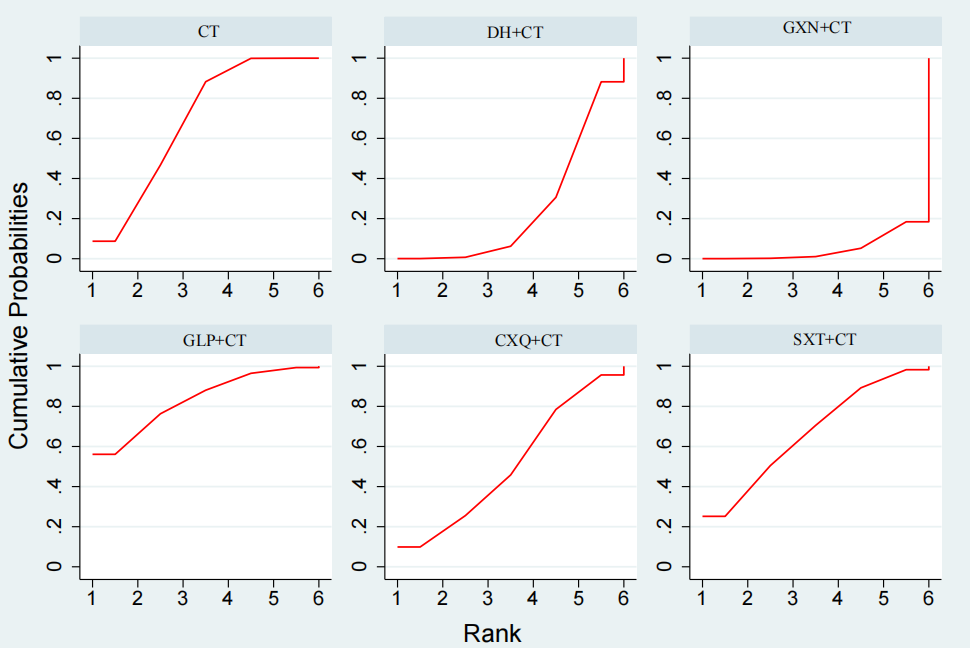
**

**E F**

**
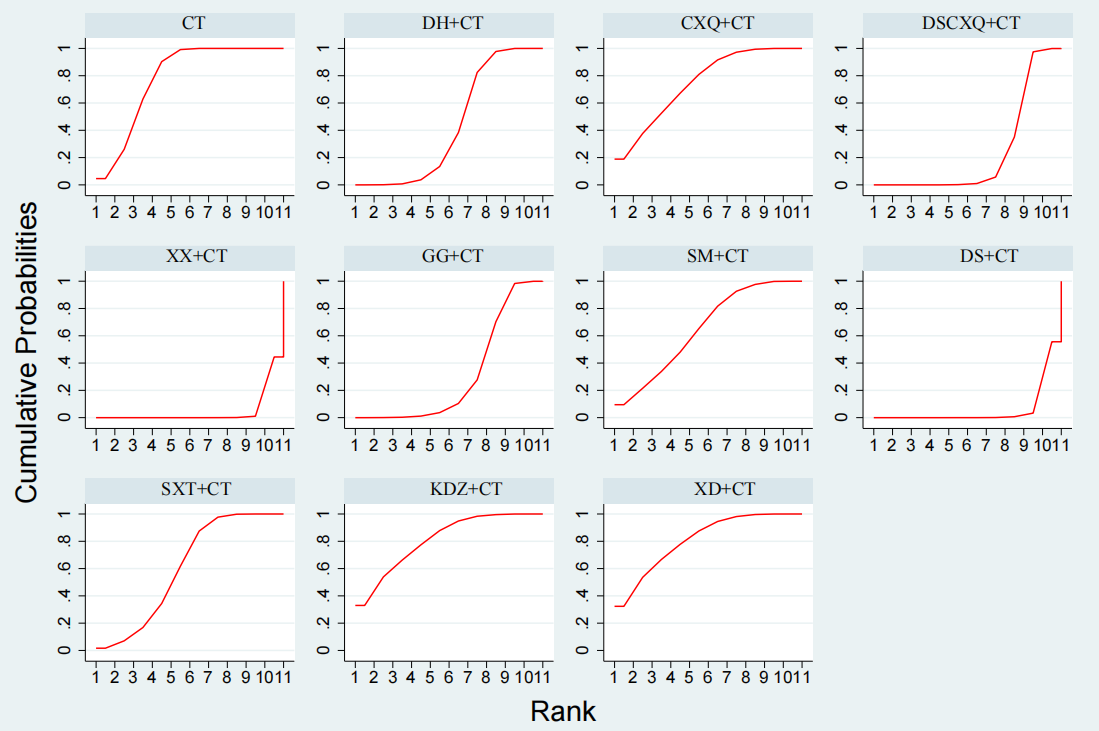
** **
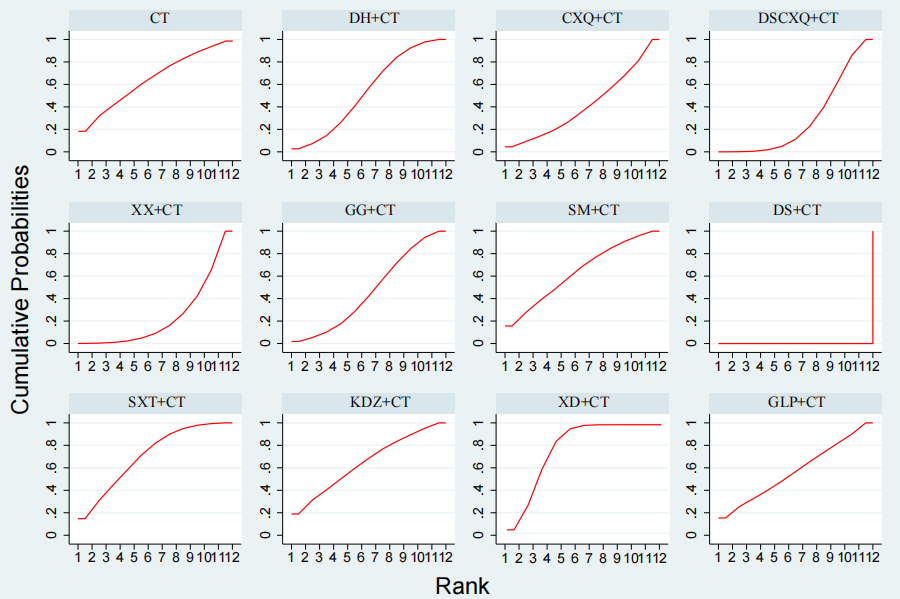
**

**G H**

**
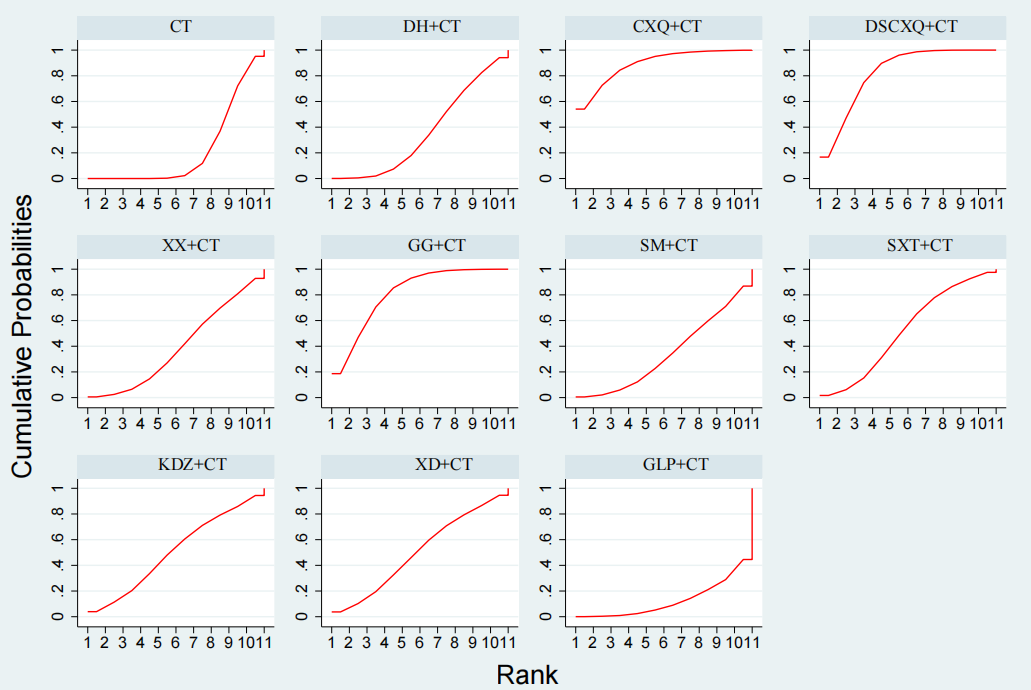
** **
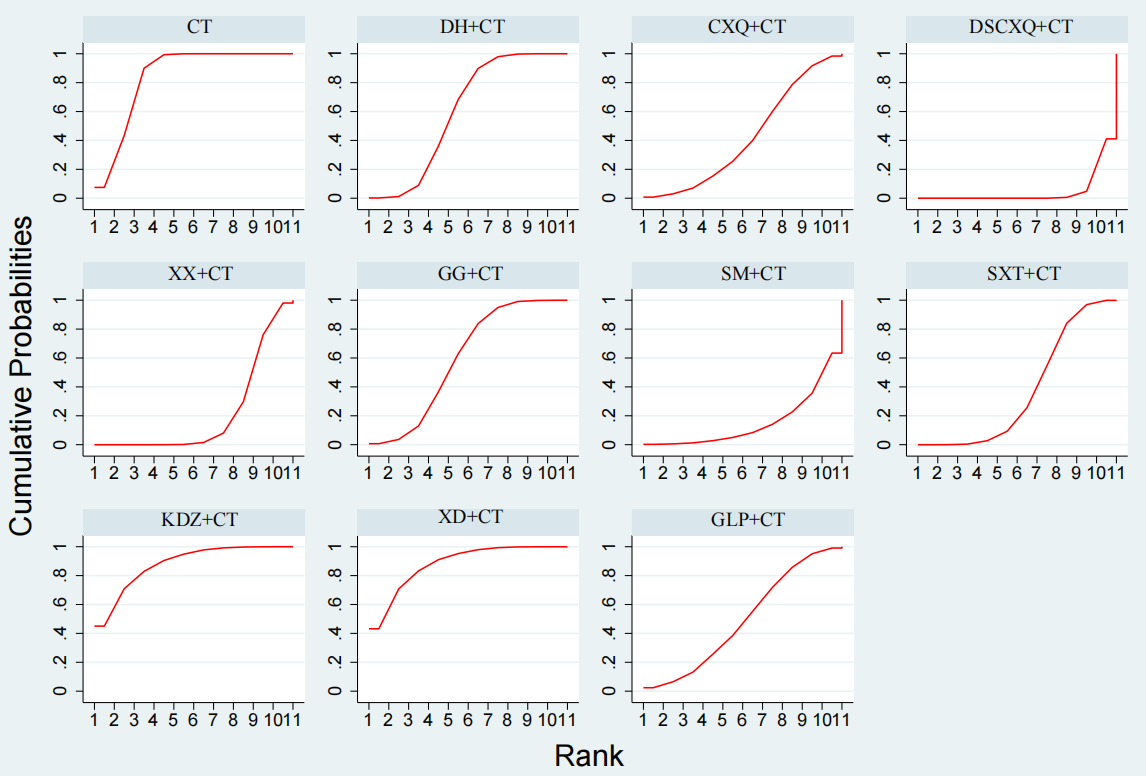
**

**I J**

**
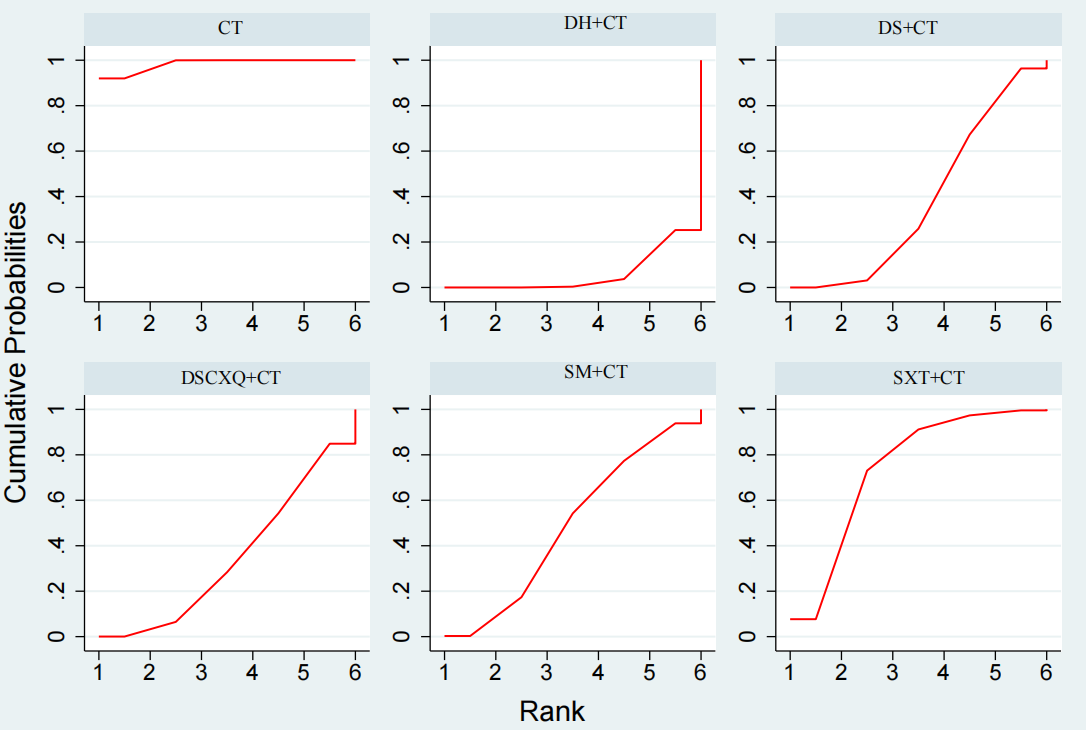
** **
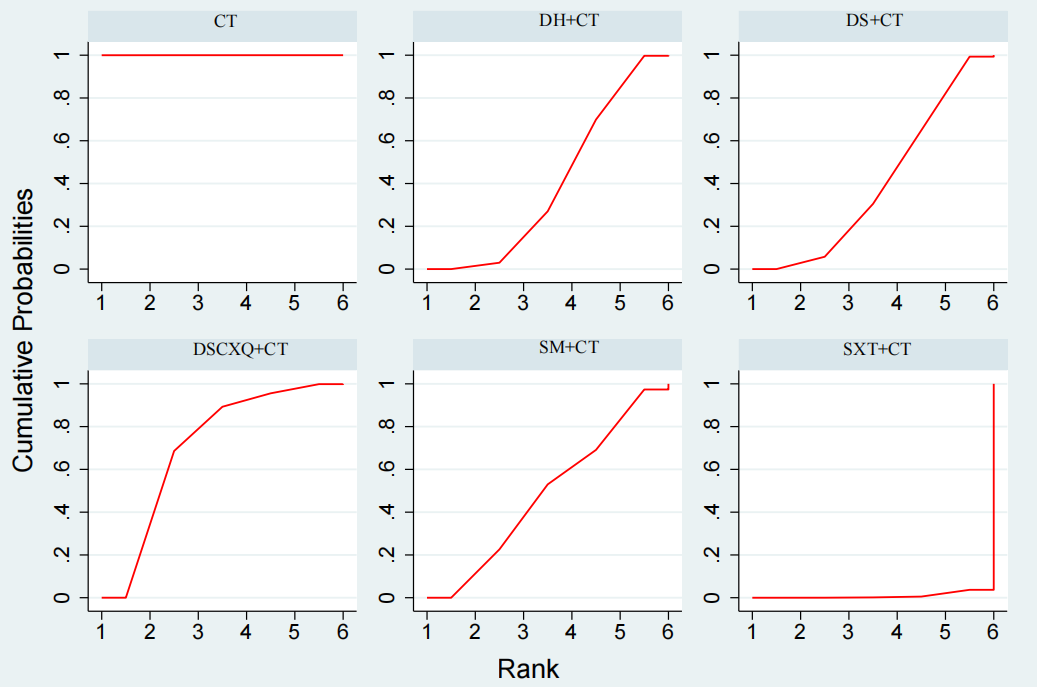
**

**K L**

***Supplementary figure S5*** Ranking probabilities of comparable treatments. **(A)** total effective rate; **(B)** EGG effective rate; **(C)** Effective rate of angina pectoris; **(D)** FBG; **(E)** PBG; **(F)** HbA1c; **(G)** TC; **(H)** TG; **(I)** HDL; **(J)** LDL; **(K)** Frequency of angina pectoris; **(L)** Duration of angina pectoris.

***Supplemantary references:***

1.Wang, N. (2021).Effects of Danhong injection combined with bisoprolol on ventricular arrhythmia in patients with type 2 diabetes mellitus complicated by coronary heart disease. Chin J Prim Med Pharm. 28 (12),1784-1788.doi: 10.3760/cma.issn1008-6706.2021.12.006.

2. Zhao, Y.H., and Han,W. (2021).Danhong injection combined with bisoprolol in the treatment of type 2 diabetes with coronary heart disease ventricular arrhythmia. Prac J Med ＆ Pharm. 38 (02), 112-116.doi:10.14172/j.issn1671-4008.2021.02.005.

3. Lin,T.M., and Hao,Q.B.(2020). Efficacy of guanxining injection for the treatment of coronary heart disease combined with type 2 diabetes in the elderly. Shenzhen Journal of integrated traditional Chinese and Western medicine. 30 (11), 27-28. doi: 10.16458/j.cnki.1007-0893.2020.11.012

4. Sun,F.Y.,Zhang,Y.N.,Wang,Y.M.,Zhao,W.J.,Zhang,S.L.(2019).Clinical Effect of Danhong Injection on Angina Pectoris of Coronary Heart Disease with Diabetes Mellitus and Heart Blood Stasis. Medical diet and health.15, 97 – 100.doi: CNKI:SUN:YXSL.0.2019-15-068.

5. Zhou, X.,Y. (2017).Treatment of 40 cases of type 2 diabetes complicated with coronary heart disease and chronic heart failure with Gualou PI injection. Henan traditional Chinese medicine. 37 (12), 2114-2117. doi: 10.16367/j.issn.1003-5028.2017.12.0726.

6. Fu,W.,J.(2017).Clinical observation of Danhong injection in treating angina pectoris of diabetes complicated with coronary heart disease. World latest medical information abstract.17 (80),66-68.doi: 10.19613/j.cnki.1671-3141.2017.80.055.

7. Pei,J.,L.(2017).Study on the effect of sodium tanshinone IIA sulfonate injection in the treatment of diabetes complicated with coronary heart disease.Contemporary Medicine.15 (17),134-135.doi:CNKI:SUN:QYWA.0.2017-17-099.

8.Chang,N.(2017). Effect analysis of tetramethylpyrazine injection in treating coronary heart disease with diabetes.Journal of Qiqihar Medical College.38 (13),1560-1561.doi: 10.3969/j.issn.1002-1256.2017.13.032.

9. Wang,J.,J.(2017).Wang Kai, Han Xuchen. Clinical efficacy of Danhong injection in treating coronary heart disease with diabetes.Liaoning Journal of traditional Chinese medicine.44 (07),1429-1430.doi: 10.13192/j.issn.1000-1719.2017.07.030.

10. Jiao,C.,J.,Yang,C.,Y.(2016).Clinical Research on Danhong Injection in Treatment of Diabetes and Heart Failure Caused by Coronary Artery Disease. Diabetes New World. 19(24),27-28.doi:10.16658/j.cnki.1672-4062.2016.24.027.

11. Guan,X.,B.(2015).Analysis of clinical effect of Salvia miltiorrhiza and tetramethylpyrazine on patients with diabetes complicated with coronary heart disease.Diabetes new world.13,15-16.doi: 10.16658/j.cnki.1672-4062.2015.13.049.

12. Xia,Z.,H., Yan,Z.,J., Han, B.(2015).Danhong Injection on the Treatment of TypeⅡDiabetes with Coronary Heart Disease for 34 Cases. Modern Distance Education of Chinese Medicine.10 (2),51-52.doi: 10.3969/j.issn.1672-2779.2015.10.027.

13.Ji,M.,C.(2015).Observation on the therapeutic effect of Dengzhanxixin injection combined with atorvastatin on type 2 diabetes complicated with coronary heart disease.Journal of modern Chinese and Western medicine.24 (13), 1432-1434.doi: 10.3969/j.issn.1008-8849.2015.13.

14. Du,X.,J.,and LI,X.,W.(2014).The clinical observation of low dose puerarin injection on the treatment of diabetes mellitus complicated with angina pectoris of coronary heart disease. Chin J of Clinical Ｒational Drug Use.7 (34),10-11.doi: 10.3969/j.issn.1674-3296.2014.34.008.

15. Liu, M. (2014).Clinical efficacy of Danhong injection in treating angina pectoris of diabetes complicated with coronary heart disease. Jilin Medical Journal.35 (31), 6984-6985.doi: 10.3969/j.issn.1004-0412.2014.31.061.

16.Hu,X.,L., and Jia,G.,L.(2014).Clinical Effects of Trimetazidine Combined with Salvia Ligustrazine Injections in the Treatment of Unstable Angina in Coronary Heart Disease with Type 2 Diabetes. China Pharmacist. 17 (4),3-5.doi: CNKI:SUN:ZYSG.0.2014-04-042.

17. Fang,C., and Wang,X.,L. (2014).Analysis of clinical effect of Salvia miltiorrhiza and Ligustrazine on 50 cases of diabetes complicated with coronary heart disease.Inner Mongolia traditional Chinese medicine. 33 (01), 28. doi: 10.16040/j.cnki.cn15-1101.2014.01.258.

18. Zhang,W.,M.(2013).Inical Observation of Danhong Injection in Treating Diabetes Mellitus Complicated with Coronary Heart Disease and Angina Pectori. Chinese medical innovation.10 (36): 18-19.doi: 10.3969/j.issn.1674-4985.2013.36.007.

19. Tan,G., and Li,M. (2013).Observation on the effect of Danhong Injection on blood glucose and other indicators in patients with diabetes and coronary heart disease. People's military medical journal. 56 (10), 1190-1191.doi: CNKI:SUN:RMJZ.0.2013-10-046.

20. Lu,A.,P.(2012).Guanxinning Injection in the treatment of 103 elderly patients with diabetes complicated with coronary heart disease and angina pectoris.Chinese Journal of Gerontology.32 (18): 4024-4025.doi: 10.3969/j.issn.1005-9202.2012.18.080.

21.Yang,T.,Y.(2012). Clinical bservation of Danhong injection in the treatment of diabetes complicated with coronary heart disease.Contemporary Medicine. 18 (7): 2.doi: 10.3969/j.issn.1009-4393.2012.7.059.

22. He,Z.(2012).Clinical ervation on 40 cases of angina pectoris in diabetes complicated with coronary heart disease treated with Danhong injection based on syndrome differentiation.China Practical Medicine.7 (2),151-152.doi: 10.3969/j.issn.1673-7555.2012.02.120.

23. Gao,Y.(2011).Observation on the curative effect of Danhong injection in the treatment of senile diabetes complicated with coronary heart disease and angina pectoris.Mdical Information: Mid term Journal.24 (8): 3990-3991.doi: 10.3969/j.issn.1006-1959.2011.08.550.

24. Li,C.,T.,Jia,X.,Z.(2011).Guanxinning Injection in the treatment of 100 cases of angina pectoris caused by diabetes coronary heart disease.Chinese Journal of Clinical Health Care.14 (3):306-307.doi: 10.3969/J.issn.1672-6790.2011.03.032.

25.Fang,W.,G.(2011). Observation on therapeutic effect of Shuxuetong Injection on diabetes complicated with coronary heart disease.Contemporary Medicine.17 (15): 68-69.doi: 10.3969/j.issn.1009-4393.2011.15.048.

26.DongH.,J.(2009).Clinical observation of Shenxiong Glucose Injection in the treatment of diabetes complicated with coronary heart disease and angina pectoris.Clinical Medicine Practice. 12 (2),2281-2282.doi: CNKI:SUN:SXLC.0.2009-35-025.

27. Wan,J.(2009).Danhong injection in the treatment of 48 cases of diabetes complicated with coronary heart disease.Jurnal of Yangtze University Natural Science Edition.6 (4),112-113.doi: 10∙3969∙j.isn∙1673-1409.

28. Xing,X.,J. and Wang,F.,Y.(2009).Observation on the therapeutic effect of Danhong Injection on diabetes complicated with angina pectoris of coronary heart disease.World Journal of Integrated Traditional and Western Medicine.9 (2),661-662.doi: 10.3969/j.issn.1673-6613.2009.09.019.

29. Xie ,W.,T.,,Xia,P.,Y., Pei,C.,C.(2009).30 cases of type 2 diabetes complicated with coronary heart disease treated with integrated Chinese and Western Medicine.Guangxi Journal of Traditional Chinese Medicine. 32 (1):11-13.doi: 10.3969/j.issn.1003-0719.2009.01.005.

30. Wei ,Y.,L. and Zhou,H.(2008).Observation on the therapeutic effect of Danshen needle plus insulin on diabetes complicated with angina pectoris of coronary heart disease. Youjiang Medical Journal. 36 (5): 543-544.doi: 10.3969/j.issn.1003-1383.2008.05.012.

31. Wu,H.,B,Liu,D.,L., Kong,H.,H.(2011).Simvastatin combined with Danshen injection in the treatment of coronary heart disease complicated with diabetes . International Medical and Health Herald. 17 (15): 1867-1869.doi: 10.3760/cma.j.issn.1007-1245.2011.15.035.

32. Sun,P.(2008).Acanthopanax senticosus treating 32 cases of angina pectoris in diabetes complicated with coronary heart disease. Journal of Modern Integrated Chinese and Western Medicine. 17 (6): 866-867.doi: 10.3969/j.issn.1008-8849.2008.06.042.

33. Huang,M.,B.(2007).Observation on the therapeutic effect of Dengzhanxixin injection on type 2 diabetes complicated with coronary heart disease.China Chronic Disease Prevention and Control.15 (6): 573-574.

34. Wang,S.,G.,Zhang,Y.,L(2007).Effect of Shuxuetong Injection on plasma ET-1, ICAM-1, TNF in patients with coronary heart disease and diabetes- α And P-selectin levels.Journal of Cardiovascular and Cerebrovascular Diseases of Integrated Traditional Chinese and Western Medicine.5 (007),574-575.doi: 10.3969/j.issn.1672-1349.2007.07.005.

35. Zeng,Z.,H., Zhou,X.,M., Zhou,W.,Z.(2007).Clinical observation of Kudiezi Injection in treating type 2 diabetes complicated with coronary heart disease. Zhejiang Clinical Medicine.9 (6): 773.doi: 10.3969/j.issn.1008-7664.2007.06.038.

36. Liao,Z.,M.(2006).Observation on the therapeutic effect of Shenmai injection combined with Ciwujia Injection on ventricular premature beats in diabetes complicated with coronary heart disease.Henan Traditional Chinese Medicine. 26 (11): 75-76.doi: 10.3969/j.issn.1003-5028.2006.11.062.

37. Du,X.,J., Li,X.,W. (2006).Observation on the clinical effect of puerarin and Erigeron breviscapus injection in the treatment of type II diabetes complicated with coronary heart disease and angina pectoris.Journal of Inner Mongolia University for Nationalities.4, 427-429.doi: 10.3969/j.issn.1671-0185.2006.04.021.

38. Hou,T.,H., Zhang ,Z., Tian,Y.,T(2003).Clinical observation of Ciwujia injection in the treatment of 36 cases of diabetes complicated with coronary heart disease.Journal of Shaanxi University of Traditional Chinese Medicine. 26 (004): 9-10.doi: 10.3969/j.issn.1002-168X.2003.04.005.

39. Li,J.,Ou,Y., F., Yang,J. (2003).Clinical observation of Xingding injection in treating type 2 diabetes complicated with coronary heart disease. Sichuan Medical Journal. 24 (2): 133-134.doi: 10.3969/j.issn.1004-0501.2003.02.011.

40. Liu,Y.,H.,Ma,J.,X., Guo,X.,Y. (2001).Analysis of 32 cases of diabetes coronary heart disease treated with Ligusticum chuanxiong injection.Journal of Changchun College of Traditional Chinese Medicine. 17 (4):12-13.doi: 10.3969/j.issn.1007-4813.2001.04.013.

41.Liu,J.,Xu,L.,Yang,X.,T.(2001).32 cases of diabetes and coronary heart disease treated by Integrated Chinese and Western Medicine. Journal of Traditional Chinese Medicine. 29 (6), 7.doi: CNKI:SUN:ZYXB.0.2001-06-004.

42. Zhang,Z.,X.(2005).Observation on the therapeutic effect of Breviscapine Injection on diabetes ischemic heart disease.Practical Clinical Practice of Integrated Chinese and Western Medicine. 5 (5), 46-46.doi: 10.3969/j.issn.1671-4040.2005.05.037.

43. Zhao,H.,L., Zhang.,Y.,A.,Sun,F.,Y.(2020).Clinical value analysis of Danhong injection in the treatment of diabetes complicated with coronary heart failure. Diabetes world.17 (4),115-116.

44. Zhang,L.(2020).Clinical efficacy of Salvia miltiorrhiza and ligustrazine in the treatment of diabetes patients with coronary heart disease.Chinese Medical Guide. 18 (3): 171-172.doi: CNKI:SUN:YYXK.0.2020-03-148.

45. Fang,R., and Li,Y.,B.(2020).Salvia Miltiorrhiza Ligustrazine Clinical Efficacy Research for the Treat ment of Diabetic Coronary Heart Disease.18 (3),42-46.doi: CNKI:SUN:YYXK.0.2020-03-148.

46. Liu,Z.,W.(2012).Clinical treatment analysis of 36 patients with coronary heart disease and diabetes treated by traditional Chinese medicine. Chinese and Foreign Health Digest. 47, 405-405.doi: 10.3969/j.issn.1672-5085.2012.47.435.

47. Wang,Q., Kuang,P.,J.(2013).Observation on the therapeutic effect of Xuesaitong on type 2 diabetes complicated with angina pectoris.China Pharmacoeconomics.5, 81-82.doi: CNKI:SUN:ZYWA.0.2013-05-037.

48. Jia,D., Wang,Y.,B.,Fan,X.(2016).Observation on therapeutic effect of Salvia miltiorrhiza polyphenolic acid salt for injection on angina pectoris complicated with type 2 diabetes.Journal of Liaoning University of Traditional Chinese Medicine. 18 (2), 22-24.doi: 10.13194/j.issn.1673-842x.2016.02.040.

49. Wu,C.,X.,Wang,X.,F., Xie,F.,(2018).Observation on the therapeutic effect of nicorandil combined with Shenmai Injection on unstable angina pectoris combined with type 2 diabetes.Medical Clinical Research. 35 (11),2215-2217.doi: 10.3969/j.issn.1671-7171.2018.11.052.

50.He,Y.,B.,Guo,X.,D.,Liu,L.,X.(2008).Effect of Shuxuetong Injection on electrocardiogram and hemorheology in patients with diabetes complicated with unstable angina pectoris.Cardiovascular and Cerebrovascular Diseases of Integrated Traditional Chinese and Western Medicine.6 (11),1373-1373.doi: CNKI:SUN:ZYYY.0.2008-11-064.
